# Differential Patterns of IgG Subclass Responses to *Plasmodium falciparum* Antigens in Relation to Malaria Protection and RTS,S Vaccination

**DOI:** 10.3389/fimmu.2019.00439

**Published:** 2019-03-15

**Authors:** Carlota Dobaño, Rebeca Santano, Marta Vidal, Alfons Jiménez, Chenjerai Jairoce, Itziar Ubillos, David Dosoo, Ruth Aguilar, Nana Aba Williams, Núria Díez-Padrisa, Aintzane Ayestaran, Clarissa Valim, Kwaku Poku Asante, Seth Owusu-Agyei, David Lanar, Virander Chauhan, Chetan Chitnis, Sheetij Dutta, Evelina Angov, Benoit Gamain, Ross L. Coppel, James G. Beeson, Linda Reiling, Deepak Gaur, David Cavanagh, Ben Gyan, Augusto J. Nhabomba, Joseph J. Campo, Gemma Moncunill

**Affiliations:** ^1^ISGlobal, Hospital Clínic - Universitat de Barcelona, Barcelona, Spain; ^2^Centro de Investigação em Saúde de Manhiça (CISM), Manhiça, Mozambique; ^3^Spanish Consortium for Research in Epidemiology and Public Health (CIBERESP), Barcelona, Spain; ^4^Kintampo Health Research Centre, Kintampo, Ghana; ^5^Department of Osteopathic Medical Specialties, Michigan State University, East Lansing, MI, United States; ^6^Department of Immunology and Infectious Diseases, Harvard T.H. Chen School of Public Health, Boston, MA, United States; ^7^Disease Control Department, London School of Hygiene and Tropical Medicine, London, United Kingdom; ^8^Malaria Vaccine Branch, Walter Reed Army Institute of Research, Silver Spring, MD, United States; ^9^Malaria Group, International Centre for Genetic Engineering and Biotechnology (ICGEB), New Delhi, India; ^10^Unité Biologie Intégrée du Globule Rouge, Laboratoire d'Excellence GR-Ex, UMR_S1134, Inserm, INTS, Université Sorbonne Paris Cité, Université Paris Diderot, Paris, France; ^11^Infection and Immunity Program, Monash Biomedicine Discovery Institute, Department of Microbiology, Monash University, Melbourne, VIC, Australia; ^12^Burnet Institute, Melbourne, VIC, Australia; ^13^Laboratory of Malaria and Vaccine Research, School of Biotechnology, Jawaharlal Nehru University, New Delhi, India; ^14^Ashworth Laboratories, Centre for Immunity, Infection and Evolution, School of Biological Sciences, Institute of Immunology and Infection Research, University of Edinburgh, Edinburgh, United Kingdom; ^15^Noguchi Memorial Institute for Medical Research, University of Ghana, Accra, Ghana

**Keywords:** Malaria, *Plasmodium falciparum*, antibody, IgG subclass, naturally acquired immunity, protection, vaccine, children

## Abstract

Naturally acquired immunity (NAI) to *Plasmodium falciparum* malaria is mainly mediated by IgG antibodies but the subclasses, epitope targets and effector functions have not been unequivocally defined. Dissecting the type and specificity of antibody responses mediating NAI is a key step toward developing more effective vaccines to control the disease. We investigated the role of IgG subclasses to malaria antigens in protection against disease and the factors that affect their levels, including vaccination with RTS,S/AS01E. We analyzed plasma and serum samples at baseline and 1 month after primary vaccination with RTS,S or comparator in African children and infants participating in a phase 3 trial in two sites of different malaria transmission intensity: Kintampo in Ghana and Manhiça in Mozambique. We used quantitative suspension array technology (qSAT) to measure IgG_1−4_ responses to 35 *P. falciparum* pre-erythrocytic and blood stage antigens. Our results show that the pattern of IgG response is predominantly IgG1 or IgG3, with lower levels of IgG2 and IgG4. Age, site and RTS,S vaccination significantly affected antibody subclass levels to different antigens and susceptibility to clinical malaria. Univariable and multivariable analysis showed associations with protection mainly for cytophilic IgG3 levels to selected antigens, followed by IgG1 levels and, unexpectedly, also with IgG4 levels, mainly to antigens that increased upon RTS,S vaccination such as MSP5 and MSP1 block 2, among others. In contrast, IgG2 was associated with malaria risk. Stratified analysis in RTS,S vaccinees pointed to novel associations of IgG4 responses with immunity mainly involving pre-erythrocytic antigens upon RTS,S vaccination. Multi-marker analysis revealed a significant contribution of IgG3 responses to malaria protection and IgG2 responses to malaria risk. We propose that the pattern of cytophilic and non-cytophilic IgG antibodies is antigen-dependent and more complex than initially thought, and that mechanisms of both types of subclasses could be involved in protection. Our data also suggests that RTS,S efficacy is significantly affected by NAI, and indicates that RTS,S vaccination significantly alters NAI.

## Introduction

Malaria caused by *Plasmodium falciparum* is a significant health problem particularly in children under 5 years old in sub-Saharan Africa, with about 219 million cases and 435,000 deaths worldwide (1). In areas of heavy and continuous transmission of *P. falciparum*, naturally-acquired immunity (NAI) to malaria is acquired with age and exposure (2). NAI is mediated by IgG antibodies mainly to antigens of the parasite asexual blood stage (BS) (2, 3) but the specific epitope targets, subclasses and effector functions have not been unequivocally defined. Elucidating these knowledge gaps is important for the development and improvement of vaccines to control and eliminate malaria. Despite the lack of a highly efficacious malaria vaccine, the most advanced product, RTS,S/AS01, is due to start implementation studies in 2019. RTS,S/AS01E has already undergone a phase 3 trial in Africa, where it showed partial protection with overall vaccine efficacy of 25.9% in infants and 36.3% in children (4). RTS,S is a self-assembling virus-like particle consisting of a recombinant protein containing part of the central tandem repeat from the *P. falciparum* circumsporozoite protein (CSP) plus epitopes from the CSP carboxy-terminal, targeting the sporozoite, and liver stages of infection, and fused to the S surface antigen of hepatitis B (HBsAg) virus and coexpressed with HBsAg alone. In the phase 3 trial, RTS,S was formulated in the AS01E liposomal adjuvant containing monophosphoryl lipid A and QS21 and was designed to induce strong anti-CSP antibody and T helper 1 cell responses (4). Although NAI is mainly directed against BS antigens, a natural response to CSP also exists (5) and immunization with irradiated sporozoites confers sterile immunity (6).

Sero-epidemiological studies usually measure total IgG, but the specific subclass is less frequently studied. The relevance of quantifying IgG subclasses relies on their different biological properties. As a consequence, differential associations of each subclass with protection may be masked when studied together. IgG1 and IgG3 are considered to be protective antibodies against *Plasmodium* spp. infection (7–11, 11–15). They are known as the cytophilic subclasses due to their high affinity for most of the Fc receptors on diverse immune cells and their function in complement fixation and opsonization (16). This gives them the ability to mediate protection against malaria through complement-mediated lysis (17) and cell-mediated mechanisms, such as opsonic phagocytosis (15, 18–20) and antibody-dependent cellular inhibition (ADCI) (21). IgG2 and IgG4 have been classically considered as non-protective antibodies against malaria (6–8, 12, 20). In contrast to the previous subclasses, IgG2 and IgG4 have low or no affinity for complement, respectively, and are known as non-cytophilic, because they poorly engage Fc receptors (16). Therefore their main function is neutralization (16). However, IgG4 has high affinity for the activating receptor FcγRI (22), which is expressed on macrophages, monocytes, activated neutrophils, eosinophils and mast cells and is regulated by exposure to cytokines (23). In addition, IgG4 has the highest affinity compared to other subclasses to the inhibitory receptor FcγRIIb (22), at moderate levels present in B cells, macrophages and basophils (23). IgG2 and IgG4 are also suggested to have higher affinity for antigens compared to IgG1 and IgG3. As a consequence, they might out-compete the cytophilic subclasses, preventing or inhibiting cell activation (24, 25).

However, not all reports are consistent with this classical view of protective cytophilic and non-protective non-cytophilic antibodies. For example, IgG2 may have cytophilic properties in individuals carrying the H131 allele of the FcγRIIa receptor, which can bind IgG2 (26). Further, IgG2 has been associated with malaria protection (27), mostly in populations that carry the H131 allele (28, 29). Interestingly, the H131 allele has decreased binding for IgG4 compared to the other allele R131. In addition, anti-CSP IgG4 showed a possible association with protection in RTS,S/AS01 vaccinated subjects (30) and a human IgG4 monoclonal antibody against *P. falciparum* sporozoite inhibited hepatocyte invasion by sporozoites *in vitro* (30). However, our recent phase 3 trial studies with RTS,S/AS01E-induced anti-CSP antibodies showed that IgG4 to CSP was not associated with protection, whereas IgG1 and IgG3 were (31).

Apart from naturally-acquired IgG responses, maternally transferred antibodies are a source of immunity during the first months of life, and they need to be taken into account when including infants in field studies of malaria immunity, particularly as baseline factors. IgG crosses the placenta, but the subclasses show differences: IgG1 is preferentially transferred, followed by IgG4, IgG3, and IgG2 (16, 32–34). Therefore, the ability of each IgG subclass to cross the placenta might also influence the susceptibility to *Plasmodium* spp in infants and should be taken into account when analyzing IgG patterns in individuals of this age.

We have previously developed and optimized assays (35, 36) to measure IgG, IgG_1−4_ to large panels of diverse antigens with different immunogenicities using the quantitative suspension array technology (qSAT). In the context of the African pediatric multicenter RTS,S/AS01E phase 3 trial for licensure (37, 38), we previously analyzed the role of IgG, IgG_1−4_, and IgM to RTS,S antigens in vaccine efficacy (31). We found that the pattern of vaccine-induced IgG subclasses for CSP was key in immunity, with IgG1 and IgG3 being protective and IgG2 and IgG4 detrimental. Here, we aimed to better understand the differential role that IgG_1−4_ subclass responses to *P. falciparum* antigens considered as targets of NAI might have in protection against clinical malaria disease in children who are naturally exposed to the parasite and who receive a primary immunization course with three doses of RTS,S/AS01E or a comparator vaccine. To this end, we analyzed at two different time points the levels of IgG subclasses to a range of RTS,S-unrelated antigens in African children from two different malaria endemic areas, evaluating the effect of age, site, and vaccination, and the association of those antibody subclasses with protection against malaria.

## Materials and Methods

### Study Design

This study was carried out in two of the seven sites included in the multicenter immunology study MAL067, ancillary to the phase 3 randomized clinical trial MAL055 (NCT00866619): Kintampo in Ghana (representative of moderate-high malaria transmission intensity [MTI]) and Manhiça in Mozambique (representative of low MTI) (39) to be able to compare the antibody responses in relation to endemicity. These two sites were chosen due to higher availability of sufficient numbers and volumes of samples from both study visits and age cohorts. Subjects were followed up by passive case detection (PCD) starting 14 days after sample collection at month (M) 3, approximately 44 days after the third dose (M2), for the subsequent 12 months, when they were censored.

Children 5-17 months and infants 6–12 weeks at recruitment with ≥150 μL plasma/serum samples available at M0 (baseline) and M3 were selected. We included 129 RTS,S/AS01E—(46 infants and 33 children in Manhiça, 26 infants, and 24 children in Kintampo) and 66 comparator—(23 infants and 15 children in Manhiça, 14 infants, and 14 children in Kintampo) vaccinated subjects from both sites (total *n* = 195). For the correlates of malaria protection and risk analysis, 78 children and infants were randomly selected from Kintampo, and 117 participants were selected from Manhiça according to a prior case-control study of cellular markers (40) and all were analyzed in a case-control design.

The study protocol was approved by the Ethics Committees from Spain, Mozambique and Ghana, and written informed consent was obtained from parents or guardians.

### Antibody Assays

qSAT was used to measure antibody responses to 35 *P. falciparum* antigens (Supplementary Table 1) applying the xMAP™ technology (Luminex Corp., Texas). Antigens were selected on the basis of profiling BS immunity, but also for effect of vaccination on PE immune responses to sporozoite (SSP2/TRAP and CelTOS) and liver stages (LSA1). Although some of the BS antigens have been characterized as markers of exposure, such as AMA1 and MSP1_42_ (Supplementary Table 1), antigen selection was primarily directed toward prominent targets of NAI, vaccine candidates or prior association with protection in sero-epidemiological studies or animal models. Additionally, several antigens were specifically included with said characteristics and limited polymorphism (e.g., Rh2, Rh4, Rh5, and EBA140). VAR2CSA, a pregnancy-specific variant of *P. falciparum* erythrocyte membrane proteins, was included as a representation of maternally-derived antibodies. qSAT assays included bovine serum albumin (BSA) and glutathione S*-*transferase (GST) coupled beads for background determination and as a control for signal from non-specific binding of *P. falciparum* GST fusion proteins, respectively. *P. falciparum* proteins were covalently coupled directly to MagPlex beads and blocked with BSA. qSAT assays were previously standardized and optimized to control for sources of variability (35, 36, 41). Briefly, antigen-coupled multiplex beads were mixed with 50 μL of test sample, negative or positive control (42, 43), at multiple dilutions (see Supplementary Materials). After incubation and washing, biotinylated secondary antibodies were added. Following streptavidin-R-phycoerythrin incubations, samples were acquired with a Luminex 100/200 analyzer and antibody levels measured as median fluorescence intensity (MFI). Data pre-processing is detailed in Supplementary Materials.

### Statistical Analysis

Comparisons of crude Ig levels across antigens and IgG subclasses were done through boxplots with geometric means, medians and interquartile ranges (IQR), by *t*-tests, and *p*-values adjusted by the Benjamini-Hochberg approach (44) considering all antigens together within each subclass. Analyses included either all subjects or separately by visit and by vaccination, and in some cases stratifying by site, by age, and by age within a site.

To evaluate factors affecting M3 Ig levels to all antigens, we fitted first univariable and next multivariable linear regression models (coefficient, 95% CI, adjusted p-values) using M3 antibody levels (log_10_ MFI) as an outcome and including the following predictors: vaccination, sex, malaria transmission season at M3, having clinical malaria episodes between M0 and M3, and baseline variables like age (in weeks), antibody levels (log_10_ MFI), hemoglobin (Hb) concentration, weight-for-age Z score (WAZ), and height-for-age Z score (HAZ). Models were also fitted separately at pre-vaccination. Malaria transmission season was defined as high between April-October for Kintampo, and November-April for Manhiça; the remaining months were defined as low transmission. The effect of baseline antibody levels (resulting from prior parasite exposure in the child and/or maternal transfer in the infants) was evaluated using the same antigen/Ig as the outcome variable at M3. The purpose of presenting the analysis of the antibodies at baseline (before vaccination) is that our prior studies showed that they have a very significant influence on the M3 antibody levels and subsequent risk of clinical malaria (31). Linearity of the associations with continuous covariates was evaluated through penalized splines in generalized additive models (GAM); variables were modeled as linear. A stepwise algorithm was used in multivariable models.

Analysis of antibody correlates of protection was based on a case-control design. The outcome was clinical malaria detected by PCD defined by fever >37.5°C with any parasitemia (without a detection limit) in the 12 months after the start of follow-up (M3 plus 14 days). Logistic regression models (odds ratio [OR], 95% CI, adjusted *p*-values) were fitted first univariable and next multivariable to obtain the effect of different predictors in the odds of having malaria. Main predictors included levels (log_10_MFI) of antibodies at M3, and increment (log_10_-transformed) of antibody levels between M0 and M3. The impact of the other covariates (same as above) on the association between antibody responses and malaria risk/protection was also assessed. The linearity of the log_10_-transformed antibody levels was evaluated when the outcome was case-control.

Multivariable models were obtained through the stepwise algorithm, R package MASS (45) and function stepAIC. Both backwards and forward methods were combined to obtain the model with the minimum akaike information criterion (AIC). All potential variables were proposed in the first step of the model, not only the ones significant in univariable analysis. Correction for multiple testing was done by Benjamini-Hochberg. In the above analyses, statistical significance was defined at the level of *p* < 0.05, and trend up to *p* = 0.1.

Finally, we performed multi-marker analysis by principal component analysis (PCA) and Partial Least Squares Discriminant Analysis (PLS-DA) using the R packages FactoMineR (46) and DiscriMiner (47), respectively. For the PCA analysis, used to reduce all antibody responses to non-correlated variables that summarize the response, we included the log_10_-transformed levels of all antigen-subclass pairs at M3 to generate the principal components. We selected the first three principal components that best explained the variance of the data and tested these components on the variables of malaria, vaccination, age and site. For PLS-DA, the response variable was clinical malaria and analysis was performed using the log_10_-transformed levels of all antigen-subclass pairs at M3. Then, we used the PLS-DA components to run logistic regressions, including also the variables of age, site and vaccine in a multivariable model. Finally, we calculated the AUC performance using the prediction of malaria outcome obtained with the PLS-DA.

## Results

### Baseline Characteristics

RTS,S and comparator vaccinees were similar with regards to baseline characteristics (age, sex, weight, height, other vaccinations, previous malaria, season, distance to health center, Hb concentration) and most of them (93%) completed the 12-month post-vaccination follow-up (31). The median time to drop out from the study was 113 (range 21–276) days, and was due mostly to early terminations due to loss to follow-up (7 subjects) or migration (4 subjects). A total of 89 malaria clinical events were recorded during the follow-up time: 60 in Kintampo (36 in RTS,S and 24 in comparators) and 29 in Manhiça (18 in RTS,S and 11 in comparators). Thirty-five clinical malaria events (39%) were registered in the children age cohort (48% in Kintampo [*n* = 29], 21% in Manhiça [*n* = 6]), and the remaining in the infant age cohort. Parasitemia of subjects who had clinical malaria was comparable between RTS,S and comparator vaccinees. The Kaplan Meier median follow-up time was 365 days (IQR = 128 to 365).

### Factors Affecting Levels of IgG Subclasses to *P. falciparum* Antigens

**Figure 1:**
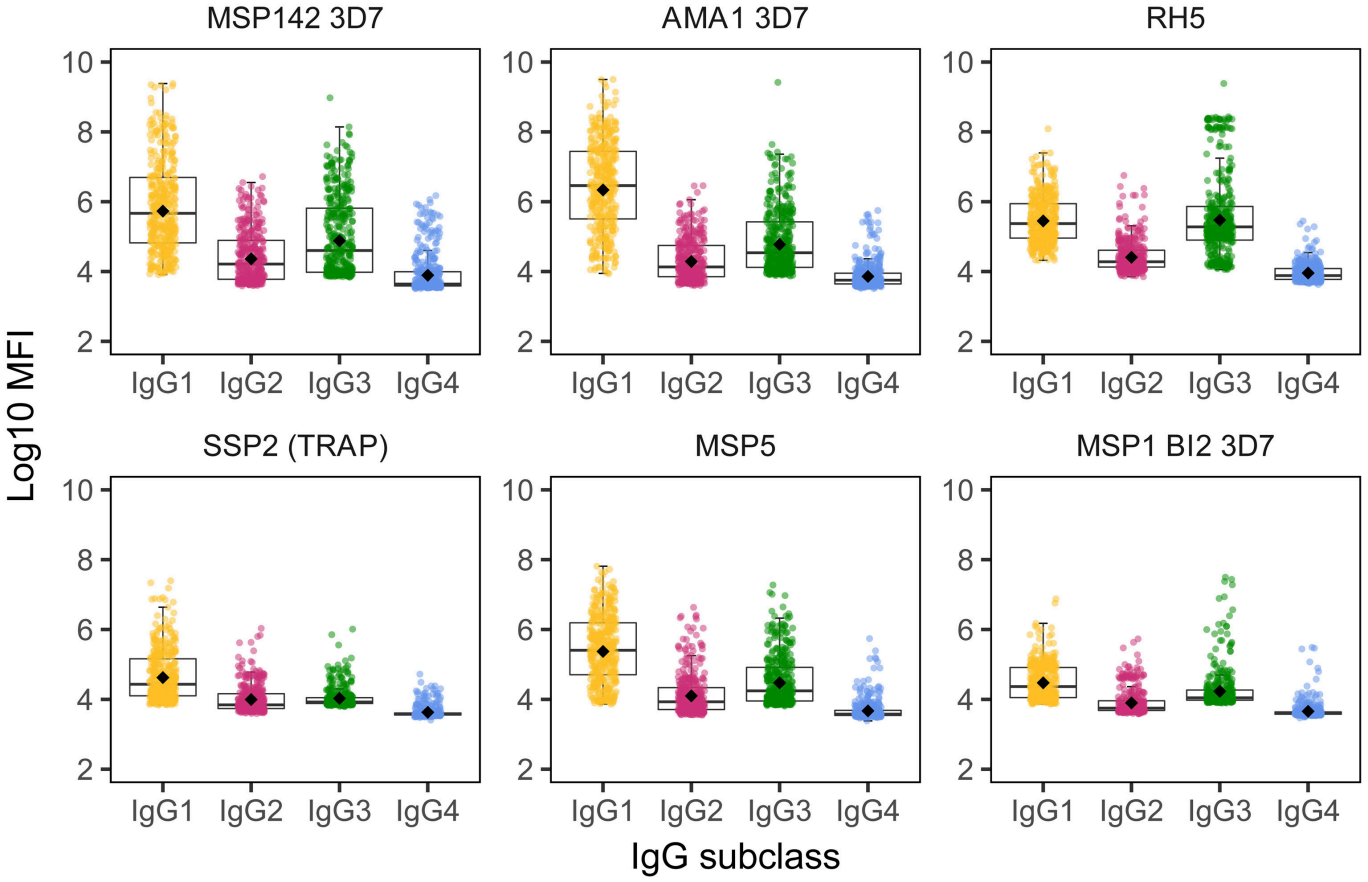
IgG subclass distribution to *Plasmodium falciparum* antigens. Representative examples from the following parasite antigens (Supplementary Table 1 for more details) whose analysis of antibody subclass responses showed differential patterns: MSP1_42_ (3D7 strain), AMA1 (3D7 strain), RH5, SSP2 (or TRAP), MSP5, and MSP1 block 2 (3D7 strain).

We detected IgG responses to most malarial antigens with a predominance of the IgG1 subclass, followed by IgG3, although some antigens (MSP2, MSP1 block [bl] 2 constructs, except the Well strain, and RH5) had similar levels of IgG1 and IgG3 (Figure 1 and Supplementary Figure 1). Levels of IgG2 were lower than those of IgG1 or IgG3, while levels of IgG4 were the lowest. The magnitude of responses at the study visits M0 and M3 (1 month post-vaccination) was differentially affected by several factors.

#### Effect of RTS,S Vaccination

**Figure 2:**
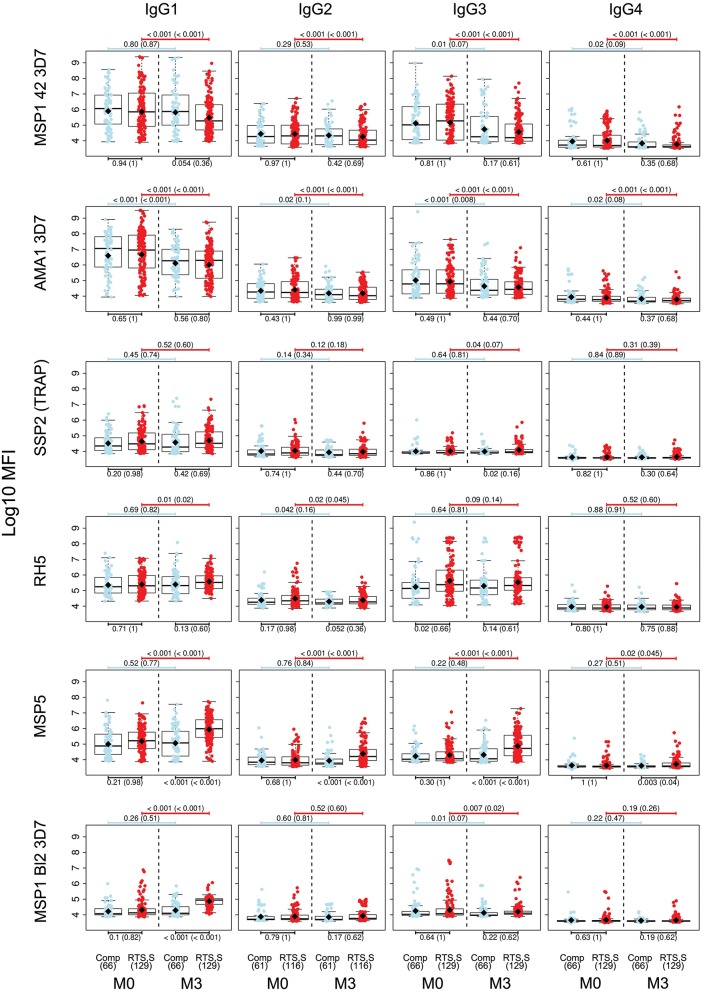
IgG subclass distribution to *Plasmodium falciparum* antigens per visit and vaccination group. Representative examples. Adjusted *p*-values are shown in parenthesis.

**Figure 3:**
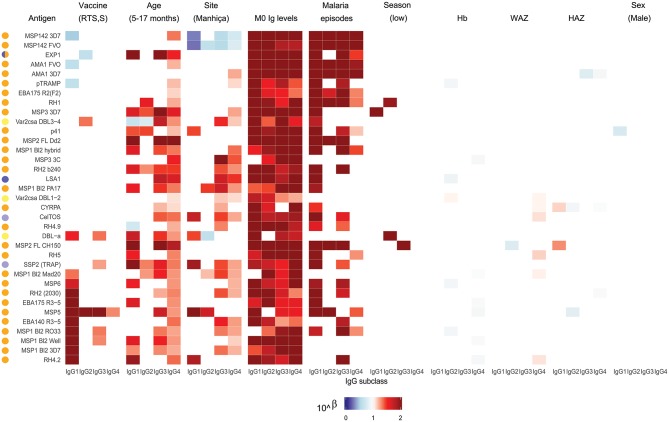
Heatmap of multivariable analysis of the effect of RTS,S/AS01E vaccination and demographic, clinical, and epidemiological variables on IgG subclass levels to *Plasmodium falciparum* pre-erythrocytic and blood stage antigens at month 3 visit. Linear regression was performed with antibody levels (log_10_MFI) as outcome and other covariates adjusted (in parenthesis the reference category). Color coded cells show the 10^∧^Beta of each predictor (columns) for each model (rows). Only results with a significant (<0.05) *p*-value are shown. Antigen colors show the *P. falciparum* life cycle stage in which they are expressed: Pre-erythrocytic (PE) antigens from sporozoite stage (light purple), PE from liver stage (dark purple), blood stage (BS) antigens from merozoite stage (orange), BS from trophozoite stage (yellow).

**Figure 4:**
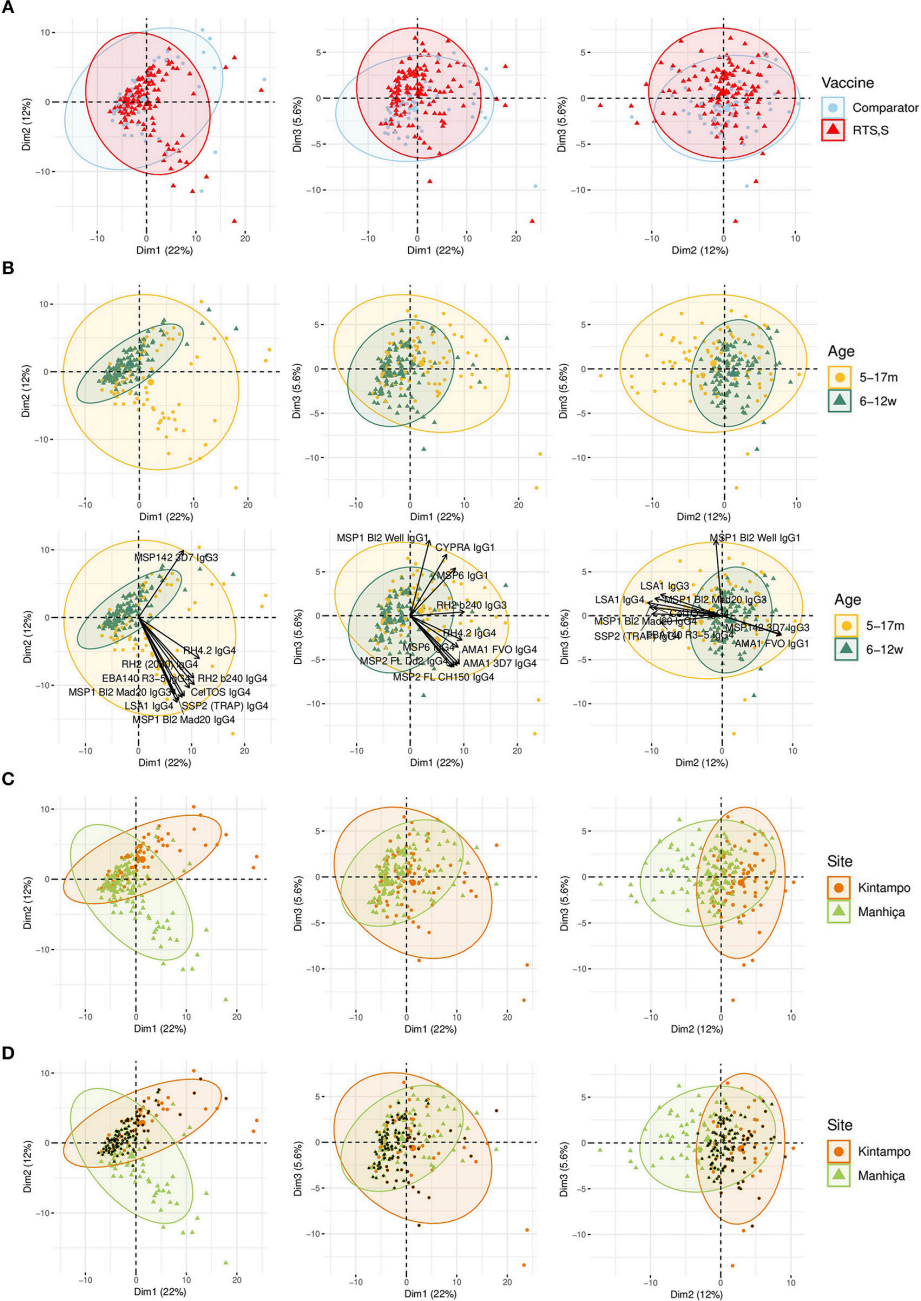
Principal Components Analysis (PCA) of the antibody responses at month 3 (M3). The plots of the distribution of individuals by type of vaccine received **(A)**, age **(B)**, site **(C)**, or site plus age **(D)** are shown. The first three principal components (Dim 1, Dim 2, and Dim 3) that explained the highest percentage of the variance of the antibody responses at M3 (percentage in parenthesis) were chosen for representation. Representation of the 10 first variables (antigen-IgG subclass pairs) that contributed to the principal components **(B)**. Black dots represent infants **(D)**.

For IgG1, three different patterns of antibody response to vaccine-unrelated antigens emerged at M3 upon RTS,S immunization compared to M0 or with comparator vaccinees (Figures 2, 3 and Supplementary Figure 2, multivariable models): decrease in antibody levels, increase in levels, and no change in levels. In multivariable models, RTS,S vaccination decreased significantly IgG1 levels to MSP1_42_ 3D7, AMA1 FVO, and pTRAMP (Figure 3). In contrast, RTS,S vaccination significantly increased IgG1 levels to DBLα, MSP1 bl2 (3D7, Well, RO33, and Mad20 strains), MSP6, RH2 2030, EBA175 R3-5, MSP5, EBA140 R3-5, and RH4.2 (trend for MSP2 CH150 and RH5). The IgG1 levels to the rest of antigens were not significantly affected by RTS,S vaccination. IgG3 antibody levels to DBLα, SSP2, MSP5, and MSP1 bl2 (Well and RO33 strains) were higher after RTS,S vaccination (trend for RH2 2030 and RH4.2), but the rest of responses did not change. IgG2 levels to EXP1 decreased after RTS,S vaccination while levels increased for VAR2CSA DBL3-4 and MSP5. The levels of IgG4 did not significantly change with vaccination except for MSP5 that were increased as with the other subclasses. PCA analysis (Figure 4A) showed that, globally, IgG subclass responses did not differ significantly by vaccination.

#### Effect of Age

**Figure 5:**
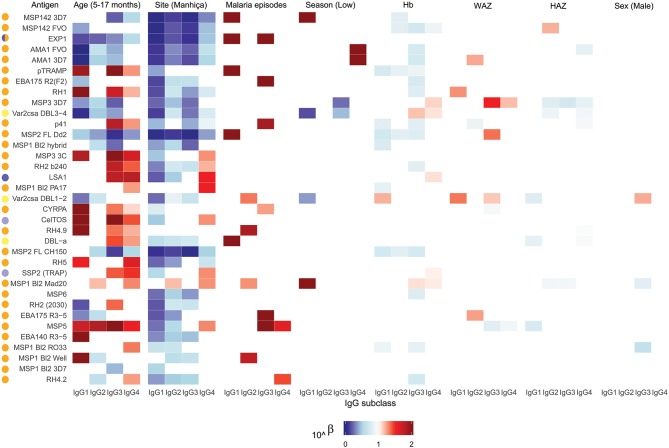
Heatmap of multivariable analysis of the effect of RTS,S/AS01E vaccination and demographic, clinical, and epidemiological variables on IgG subclass levels to *Plasmodium falciparum* pre-erythrocytic and blood stage antigens at baseline. Linear regression was performed with antibody levels (log_10_MFI) as outcome and other covariates adjusted (in parenthesis the reference category). Color coded cells show the 10^∧^Beta of each predictor (columns) for each model (rows). Only results with a significant (<0.05) *p*-value are shown. Antigen colors show the *P. falciparum* life cycle stage in which they are expressed: Pre-erythrocytic (PE) antigens from sporozoite stage (light purple), PE from liver stage (dark purple), blood stage (BS) antigens from merozoite stage (orange), BS from trophozoite stage (yellow).

Comparing antibody profiles over time, IgG levels significantly decreased from M0 to M3 for some antigens probably reflecting the decay of maternal antibodies, while for others there was a statistically significant increase and others did not show differences (Supplementary Figure 3). Notably, for most antigens, the increase was mainly observed in RTS,S vaccinees, confirming the effect of vaccination described above. In multivariable analysis, the effect of age group on baseline levels of IgG1 and IgG3 had a mixed pattern. Levels against 11 of 35 antigens were significantly higher in infants than children, while levels against 10 and 13 out of 35 antigens were significantly higher in children than infants for IgG1 and IgG3, respectively (Figure 5). These latter antigens were pTRAMP, RH1, MSP3 3C, CyRPA, CelTOS, RH4.9, and MSP5 for both subclasses, RH5, EBA140, MSP1 bl2 Well for IgG1, and p41, RH2 b240, LSA1, DBLα, and SSP2 for IgG3. Levels of IgG2 were generally lower in children than infants (12 out of 35 antigens) or not different except for MSP5 and MSP2 bl2 Mad20, for which IgG2 levels were significantly higher in children than infants (Figure 5). In contrast, IgG4 levels to 17 of 35 antigens were significantly higher in children than infants, except for MSP1_42_ 3D7, EXP1, and MSP2 for which levels were significantly lower in children. In multivariable models adjusted by M0 IgG levels and vaccination, among other variables, M3 IgG levels were generally higher in children than infants or not significantly different, except for IgG1 to RH4.9 and IgG1 and IgG2 to VAR2CSA DBL3-4 that were lower (Figure 3). These M0 and M3 results could be observed also in univariable analyses (Supplementary Figure 4). The higher baseline IgG1 and IgG3 levels against many antigens in infants than children suggest maternally-transferred antibodies, whereas IgG4 antibodies appeared to be poorly transferred and/or already induced in the first months of life. Higher levels in children than infants at M3 adjusting for baseline levels suggests higher acquired responses in children than infants during the 3-month period. Analysis of IgG responses by PCA at M3 (Figure 4B) showed that infants clustered together while children were more disperse. The variables that contributed the most to PC1 and PC2 and contributed to segregate partially the responses that corresponded to children and infants were, on one hand, IgG3 to MSP1_42_ 3D7 and, on the other hand, IgG3 to MSP1 bl2 Mad20, and IgG4 to RH2 b240, LSA1, CelTOS, SSP2, RH2 (2030), MSP1 bl2 Mad20, EBA140 R3-5, and RH4.2.

#### Effect of Site (MTI)

In general, antibody levels were higher in Kintampo (high MTI) than in Manhiça (low MTI) for most antigens (Supplementary Figure 5). At M0, antibody levels were significantly higher in Kintampo than in Manhiça to most antigens except for IgG2 to MSP1 bl2 Mad20 and IgG4 to MSP3 3C, RH2 b240, LSA1, MSP1 bl2 (PA17 and Mad20) CelTOS, SSP2, and MSP5, which were significantly higher in Manhiça than Kintampo (Figure 5 and Supplementary Figures 6, 7). In contrast, at M3 a more mixed pattern was observed in models adjusted by baseline levels and vaccination, among other variables. Some responses did not significantly differ by site (27/35 antigens for IgG1 and 30/35 antigens for IgG2), others were higher in Manhiça than Kintampo (6/35 antigens for IgG1, 3/35 for IgG2, 15/35 for IgG3 and 19/35 for IgG4), and only anti-MSP1_42_ antibodies of all subclasses were lower in Manhiça than Kintampo (Figure 3 and Supplementary Figures 6, 7). Thus, children and infants in Kintampo had significantly higher levels to all subclasses and antigens with the exception of IgG4 against some antigens, which were significantly higher in Manhiça, but Manhiça subjects had increased levels of the other subclasses from M0 to M3 to similar or higher levels than Kintampo subjects. Analysis by PCA at M3 (Figure 4C) showed clustering of individuals by site, although there was some overlap with age. Most infants from both sites clustered together whereas PC2 discriminated children from each site (Figure 4D).

#### Effect of Other Variables

At baseline there were not very generalized or consistent significant associations with antibody levels and the rest of covariates in multivariable models (Figure 5). Hb concentrations were negatively associated with IgG1, IgG2, and IgG3 levels to some antigens but positively associated with IgG4 levels. For WAZ there were generally significant positive associations while for HAZ there were significant negative associations, markedly for IgG4. Sex was not significantly associated with M0 levels except for IgG3 to MSP1 bl2 RO33 and Mad20 (females > males) and VAR2CSA DBL1-2 (males > females). At M3, the strongest and more consistent associations were that higher M0 antibody levels and prior malaria episodes were significantly associated with higher M3 antibody levels (Figure 3).

### Antibody Correlates of Malaria Protection

**Figure 6:**
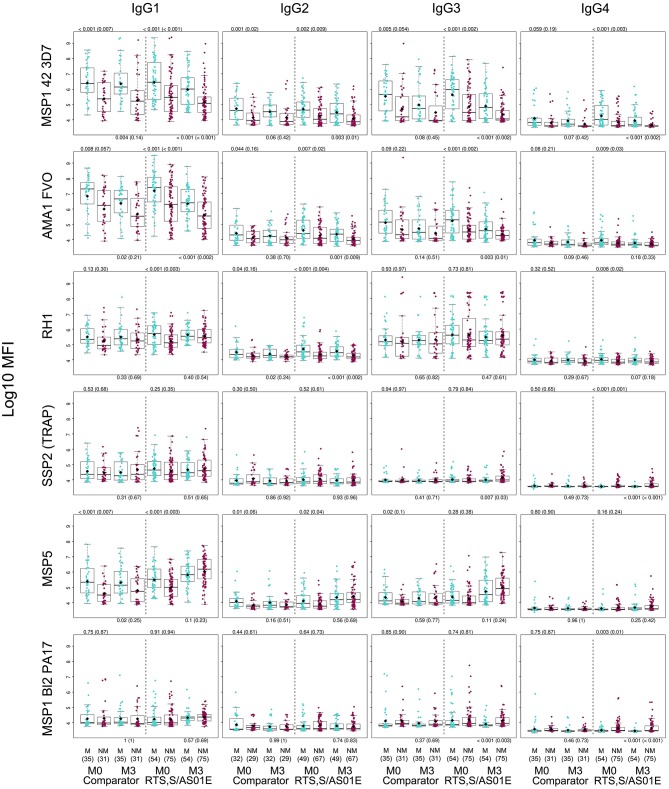
Association between month 3 IgG levels and malaria protection after RTS,S vaccination. Only representative examples are included. Adjusted p-values are shown in parenthesis. M, Malaria, NM, No malaria.

Baseline antibody levels of most subclasses/antigens were generally significantly higher in those subjects who had clinical malaria during the 12-month follow up after M3 than in those who did not (Figure 6 and Supplementary Figure 8). At M3, IgG1, IgG2 and IgG3 antibodies were also higher in malaria cases for some antigens whose levels decreased from M0 to M3 (e.g., AMA1, MSP1_42_). Stratified by age, this was more significant in children (Supplementary Figure 9). Stratified by site, these differences were not detected (Supplementary Figure 10). In contrast, M3 IgG1, IgG2, and IgG3 levels that increased after RTS,S vaccination or to those antigens whose antibodies did not decrease from M0 to M3, did not generally differ in cases and controls (Figure 6 and Supplementary Figures 8–10). IgG4 followed a different pattern; crude levels of antibodies to pre-erythrocytic (PE) antigens SSP2, LSA1, and CelTOS and to MSP1 bl2 constructs were higher in protected than non-protected subjects, not only at M3 but also at M0 (Figure 6 and Supplementary Figures 8). M3 IgG3 levels to these antigens had a similar pattern but associations were weaker. This IgG4 pattern was mainly observed in children (Supplementary Figure 9) and in Manhiça (Supplementary Figure 10). Stratified by age and site, M3 crude IgG4 (and IgG3) levels to the above and additional antigens were lower in Manhiça children with malaria after RTS,S vaccination than non-malaria controls, while the opposite pattern was seen for comparator vaccinees (Supplementary Figure 11), suggesting an interaction with vaccination (evident in this particular group).

Analysis of the change in IgG1 and IgG3 levels from M0 to M3 revealed significantly higher increments in controls than malaria cases to most of the antigens that had increased antibody responses in RTS,S vaccinees, particularly MSP1 bl2 constructs and MSP5 (Supplementary Figure 12A). Stratifying by age and site, the association was mostly seen for children and Kintampo (Supplementary Figure 12B).

**Figure 7:**
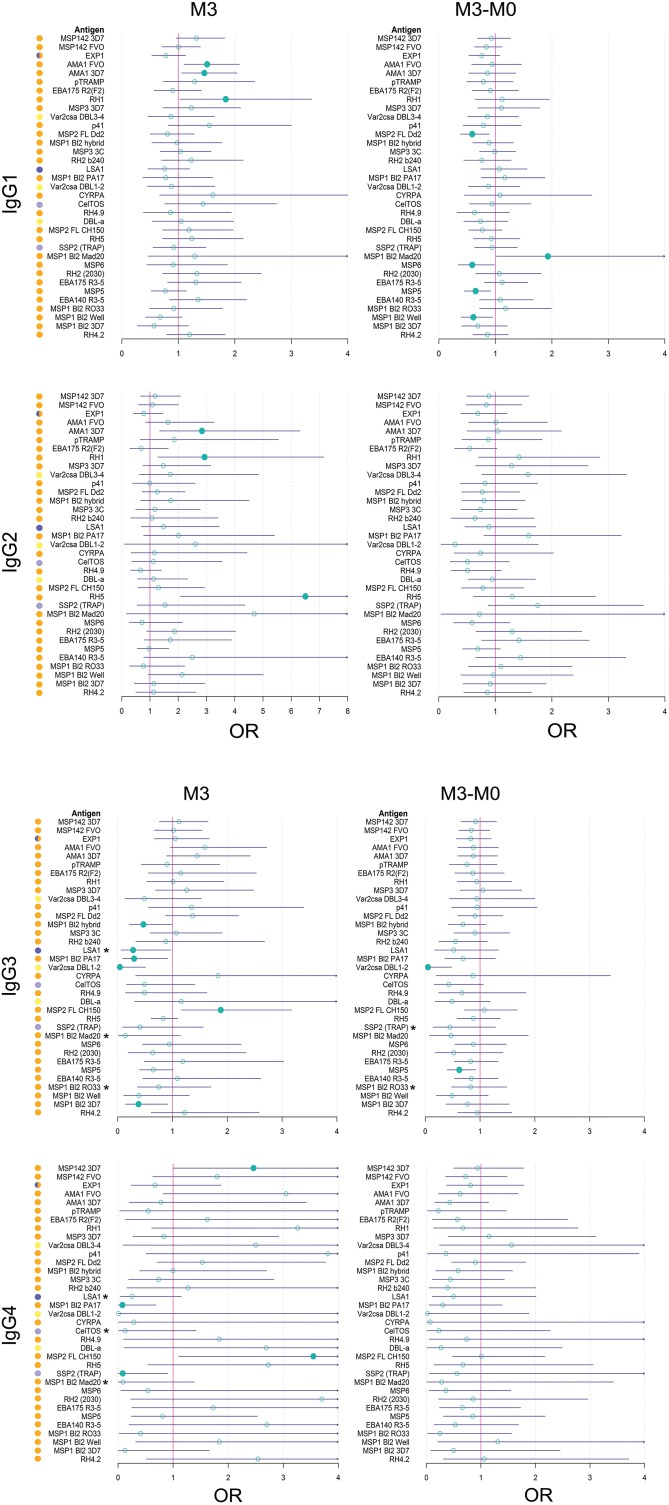
Forest plots of multivariable analysis of the association between IgG subclass responses to *Plasmodium falciparum* pre-erythrocytic and blood stage antigens, and malaria protection. Logistic regression models with antibody levels at month 3 (M3), or change in M3 and M0 levels (M3-M0), as predictors and clinical malaria as outcome, adjusted by covariates that entered the models according to the minimum akaike information criterion (RTS,S vaccination, age cohort, site, M0 antibody levels, WAZ and prior malaria episodes, depending on the antigen/subclass). OR: Odds ratio with 95% confidence intervals (CI). OR with a CI below (protection) or above (risk) 1 are represented by a colored circle. Associations with a CI below or above 1 (asterisks) in stratified analysis including only RTS,S vaccinees: IgG3 to LSA1 at M3: *OR* = 0.13 (0.02;0.71), *p* = 0.03; IgG3 to MSP1 bl2 Mad20 at M3: *OR* = 0.01 (0;0.33), *p* = 0.02; IgG3 to MSP1 bl2 RO33 at M3: *OR* = 0.33 (0.09;0.99), *p* = 0.056; IgG3 to SSP2 (TRAP) at M3-M0: *OR* = 0.23 (0.05;0.86), *p* = 0.04; IgG3 MSP1 bl2 RO33 at M3-M0: 0.47 (0.2;0.99), *p* = 0.053; IgG4 LSA1 at M3: *OR* = 0.07 (0;0.65), *p* = 0.058; IgG4 to CelTOS at M3: *OR* = 0 (0;0.28), *p* = 0.03; IgG4 to MSP1 bl2 Mad20 at M3: *OR* = 0 (0;0.25), *p* = 0.055.

In logistic regression models adjusted by vaccine, age, site and M0, among other variables when relevant, M3 IgG1 to AMA1 and RH1, IgG2 to AMA1 3D7, RH1 and RH5, and IgG3 and IgG4 to MSP2 FL CH150, were associated with increased malaria risk (Figure 7). On the contrary, levels of M3 IgG3 to MSP1 bl2 3D7, PA17, and hybrid, VAR2CSA DBL1-2 and LSA1, and IgG4 to MSP1 bl2 PA17 and SSP2, were associated with malaria protection. Also, in multivariable logistic regression models, the difference between M3 and M0 levels for IgG1 to MSP5, MSP6, MSP1 bl2 Well and MSP2 FL Dd2, and IgG3 to VAR2CSA DBL1-2 and MSP5, was associated with protection, while IgG1 to MSP1 bl2 Mad20 was associated with risk (Figure 7). In stratified analysis including only RTS,S vaccinees, IgG3 and IgG4 levels to the PE antigens LSA1, SSP2 and CelTOS and MSP1 bl2 constructs were associated with protection. Among covariates retained in multivariable models (Figure 7), RTS,S vaccination, children cohort (mostly), Manhiça site (always), low baseline antibody levels and high WAZ (specially for IgG2), were associated (statistically significantly or not) with less risk of malaria. Sex, prior malaria episodes, season, HAZ or Hb were not significantly contributors to the models, except for previous malaria that improved the IgG3 model (M3 levels, RTS,S vaccinees only) for MSP1 bl 2 constructs.

**Figure 8:**
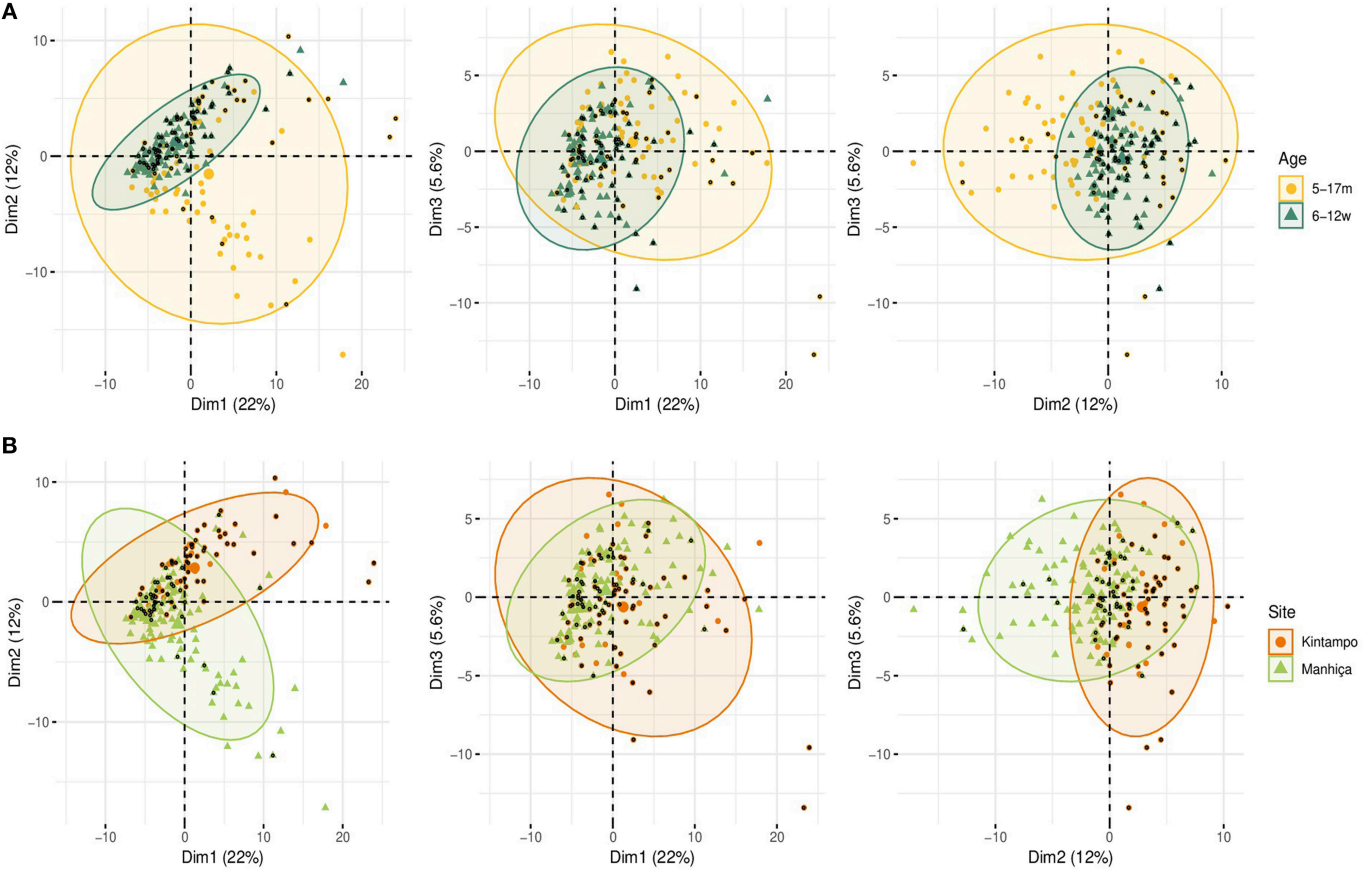
Principal Components Analysis (PCA) of the antibody responses at month 3 (M3). The plots of the distribution of individuals by age **(A)** and site **(B)** plus malaria are shown. The first three principal components (Dim 1, Dim 2, and Dim 3) that explained the highest percentage of the variance of the antibody data at M3 (percentage in parenthesis) were chosen for representation. Black dots represent malaria cases.

In PCA analysis at M3, simultaneous representation of malaria cases by age and site (Figures 8A,B) showed that most malaria cases overlapped with infants and Kintampo, respectively. This is in agreement with the observation in multivariable logistic regression models, where children and Manhiça were associated with less risk of malaria.

**Figure 9:**
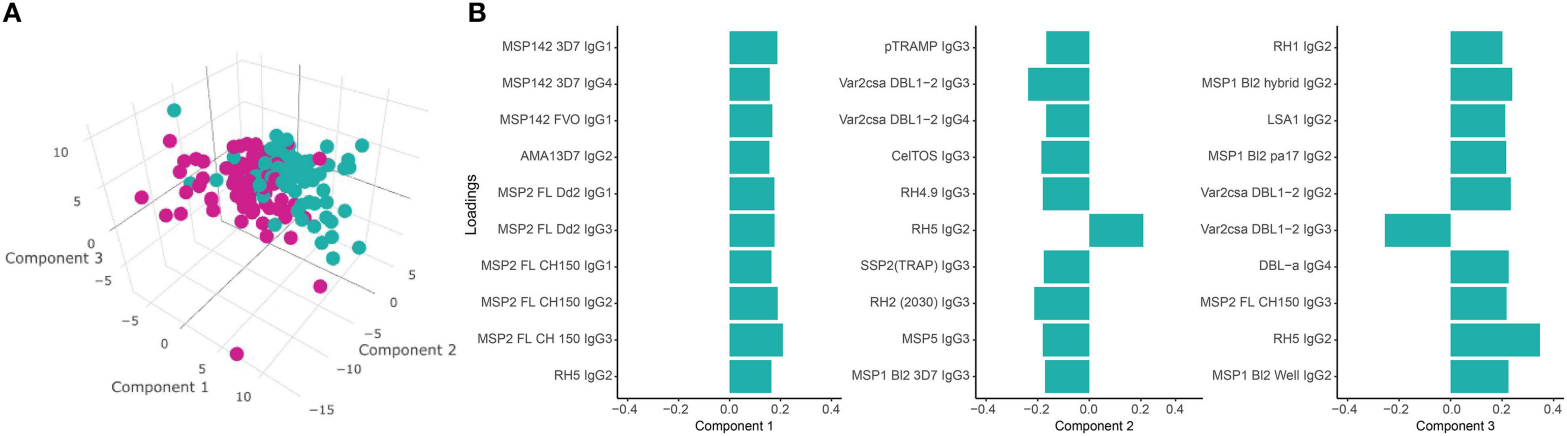
Partial Least Squares Discriminant Analysis (PLS-DA) plots. **(A)** 3D scatter-plot with the three main PLS-DA components that best enable discrimination between malaria cases (M) and non-malaria controls (NM) using antibody levels at month 3 (M3) (cumulative *R*^2^ = 0.37). **(B)** Loadings of antigen-subclass pairs with the 10 highest absolute scores for each PLS-DA component at M3.

PLS-DA at M3 with malaria as a response variable (Figure 9A) identified three main components associated with malaria. The IgG subclass responses that contributed more to these components (the 10 responses with higher loadings in each component) included IgG1 to MSP1_42_ and MSP2; IgG2 to AMA1, MSP2, RH5, MSP1 bl2 constructs, LSA1, VAR2CSA DBL1-2, RH1; IgG3 to MSP2, pTRAMP, VAR2CSA DBL1-2, CelTOS, RH4.9, MSP5, RH2 (2030), SSP2 and MSP1 bl2 constructs; and IgG4 to MSP1, VAR2CSA DBL1-2 and DBLα (Figure 9B). In a multivariable logistic model including these three PLS-DA components and adjusted by age, site and vaccine, the PLS-DA components were independently associated with malaria (component 1 [*p* < 0.001], component 2 [*p* = 0.01] and component 3 [*p* < 0.001]), while age (*p* = 0.76), site (*p* = 0.29), and vaccine (*p* = 0.57) were not. The predictive ability of the model was very high (75 out of 79 malaria cases and 94 out of 96 controls correctly predicted) with an AUC of 0.964.

**Figure 10:**
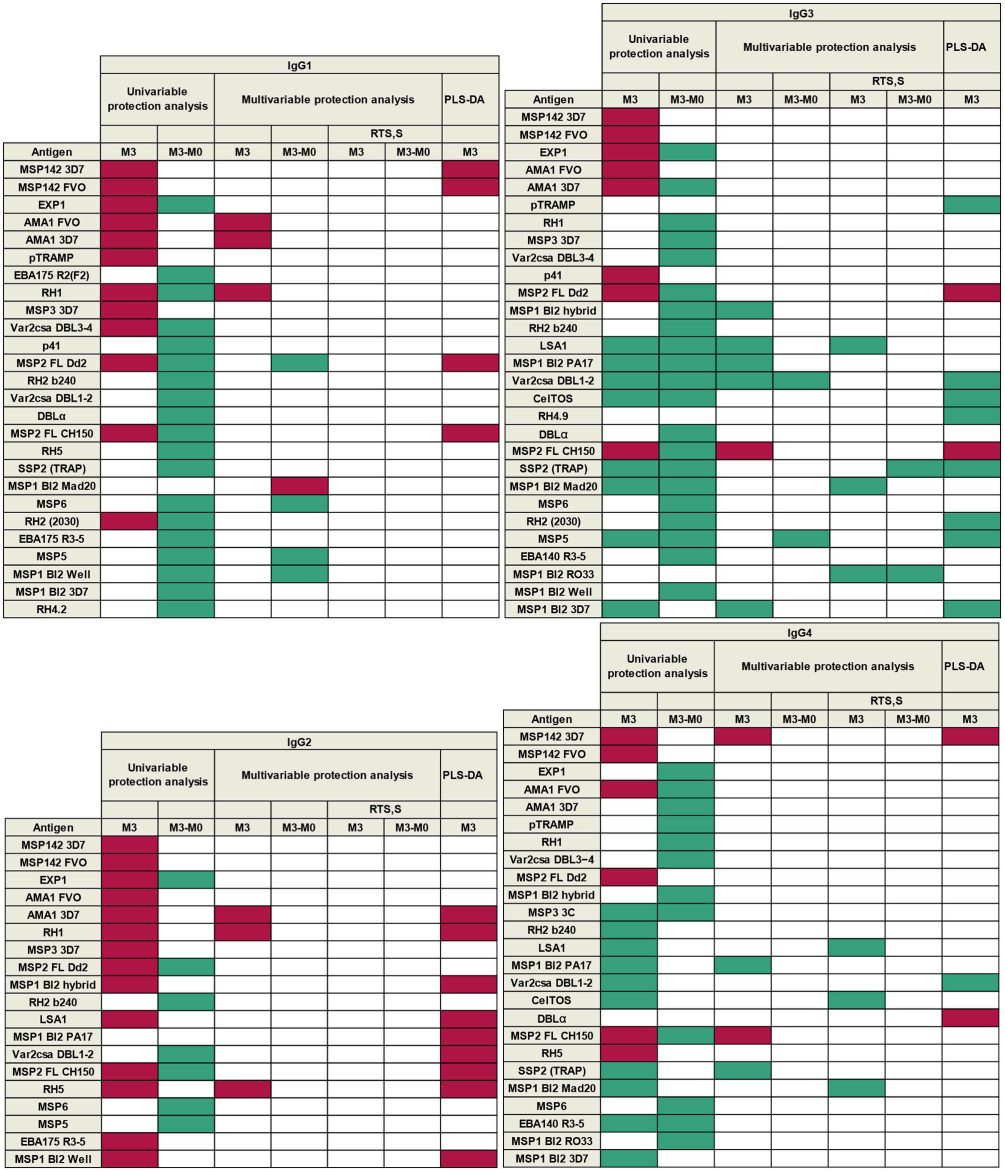
Summary of associations between antibody responses and malaria protection. Purple represents association with malaria risk and green with protection.

A summary of all results according to single-marker (univariable and multivariable regression models) and multi-marker (PLS-DA) analysis, is presented in Figure 10. This shows the groups of antigens whose levels of antibodies at M3 and/or changes in levels from M0 to M3, were associated with malaria protection (green) or risk (purple), for each IgG_1−4_ subclass response.

## Discussion

Our study found that malaria protective immunity is mostly associated with the cytophilic subclasses IgG1 and IgG3 and, in spite of the low levels, with IgG4, to certain PE and BS *P. falciparum* antigens, whereas IgG2 responses were mostly associated with malaria risk. As expected, IgG1 followed by IgG3 were the predominant subclasses in most antigens, with lower IgG2 and IgG4 levels. Consistent with previous reports, MSP1 bl2, MSP2, and RH5 predominantly induced IgG3, or similarly high IgG3 and IgG1 levels, and we found that this was also the case for DBLα (48–51). Each IgG subclass and antigen pairs studied showed a different pattern, magnitude, and association with malaria protection, with different factors affecting the outcome. Despite this complexity, we found novel and consistent results regarding determinants of the antibody response and impact on malaria outcomes.

IgG1 and IgG3 were differently associated with malaria protection, depending on the antigen specificity. IgG1 levels to antigens that are considered markers of exposure (MSP1_42_, AMA1, EXP1, etc.) were higher in infants than children and in malaria cases than controls. This could be explained by presence of maternal antibodies, which are preferentially of the IgG1 subclass due to the differential placental transfer (16, 32, 33)—as shown by VAR2CSA antibodies that should be transferred by the mother- and/or a higher pre-exposure to the parasite in these individuals. It has been previously established that a strong risk factor for future malaria is having had malaria in the past (13) which can be manifested by higher baseline levels of IgG1 to these BS antigens (52). In contrast, change from M0 to M3 in IgG1 levels to a different group of antigens (MSP5, MSP6, RH4.2, MSP1 bl2, etc.) was associated with protective responses. In the case of IgG3, levels of antibodies to SSP2, LSA1, and MSP1 bl2 at M3 were associated with less malaria risk.

There are differences between IgG1 and IgG3 that might have implications for protection and that could explain why they differ in levels, even though they show similar antigen reactivity. In terms of similarities, they both respond mainly to proteinaceous antigens and are cytophilic. However, IgG3 has a shorter half-life (7 days) compared to the other subclasses (21 days) (16, 25), meaning that the presence of IgG3 could depend on a continuous or repeated exposure to compensate for the shorter half-life, although some IgG3 allotypes have also shown extended half-lives. Therefore, malaria exposure may influence IgG subclass balance, such that early in infection an IgG1 response may be dominant, and for some antigens this may then evolve to IgG3. In addition, it is suggested that IgG3 has lower antigen-affinity than IgG1, but it activates complement more effectively and has higher affinity to Fcγ receptors (16, 25), thus, when present, it is probably more effective in mediating effector mechanisms.

We hypothesized that while cytophilic antibodies IgG1 and IgG3 would be associated with protection, non-cytophilic antibodies IgG2 and IgG4 would be associated with risk, as reported in other studies (7–11, 11–15, 53), although some studies have found protective associations with IgG2 (54). Unexpectedly, IgG4 showed potential associations with immunity despite presenting the lowest levels. In univariable analysis we found that higher IgG4 levels to all PE antigens and MSP1 bl2 constructs correlated with malaria protection. Interestingly, these responses helped discriminate between children from Manhiça and infants and children from Kintampo, suggesting that these responses may be particularly acquired in Mozambique. In multivariable analysis, higher IgG4 levels to the PE antigens SSP2 and LSA1 and one of the MSP1 bl2 constructs were associated with protection. Regarding IgG2, responses to AMA1, RH1 (like IgG1) and RH5, positively correlated with malaria risk. Consistently, in multi-marker analysis IgG2 to AMA1, RH1, and RH5 were identified as significant variables predicting malaria disease, in addition to MSP2 FL CH150, LSA1, VAR2CSA DBL1-2, and MSP1 bl2 constructs. This is in agreement with the notion that production of non-cytophilic IgG2 elicited by natural exposure to the parasite is non-protective against malaria (7–9, 13, 53). Consistent with this, we have previously found that higher levels of IgG2 to CSP induced by RTS,S vaccination are associated with malaria risk in the phase 3 trial (31). Interestingly, infants had higher IgG2 levels than children (except for MSP5 and MSP1 bl2 Mad20) at baseline, which is surprising because this subclass is considered to be the least efficiently transferred during pregnancy (16, 32, 33). This probably reflects very high maternal IgG2 levels, as shown for VAR2CSA antibodies.

Cytophilic subclasses are well known for mediating protection in malaria through their ability to fix complement (17) and mediate opsonic phagocytosis (15, 18–20) and ADCI (21). A previous observation that phagocytic activity was lower in protected than non-protected subjects vaccinated with RTS,S/AS01 (55) led to the discovery that the neutralizing and/or inhibitory function of IgG4 to CSP is associated with reduced phagocytic activity, linking IgG4 with a possible protective function in RTS,S vaccinees (30). IgG4 has the highest affinity to antigens compared to the other IgG subclasses, which could result in the ability to out-compete IgG1 and IgG3 even at low concentrations (24, 25) and may explain the different associations with risk and protection. Thus, IgG4 would be protective by blocking opsonic phagocytosis mediated by cytophilic subclasses, which could be used by *Plasmodium* spp. as a means to shelter in phagocytes and then evade the immune system (30). In view of these results, we propose that this mechanism of IgG4 protection could apply to other non-vaccine related antigens and that protection needs a delicate balance combining the cytophilic activity elicited by IgG1 and IgG3 with the blocking activity of IgG4. This balance can be affected by factors such as the target antigen, age, MTI and RTS,S vaccination (56).

Unexpectedly, IgG1 and IgG3 levels to some antigens (DBLα, EBA140 R3-5, EBA175 R3-5, MSP1 bl2 [3D7, Well, RO33, and Mad20 strains], MSP5, MSP6, RH2 2030, RH4.2, and SSP2) increased after RTS,S vaccination. In contrast, levels of IgG1 to exposure antigens (AMA1 and MSP1_42_) decreased following RTS,S immunization, suggesting that RTS,S vaccinees were less exposed to the parasite due to vaccine efficacy. Importantly, among the antigens to which antibodies were higher after RTS,S vaccination, MSP1 bl2 Well, MSP2 FL Dd2, MSP5, and MSP6 showed higher levels in controls than in malaria cases and were associated with protection in adjusted models for IgG1 and some for IgG3, while IgG1 to AMA1 and RH1, which decreased or did not change with RTS,S vaccination, were associated with malaria risk. Similarly, higher M0 to M3 change of IgG3 levels to MSP1 bl2 and MSP5, which were increased by RTS,S vaccine, was observed in controls than malaria cases. In adjusted models, IgG3 to MSP1 bl2, MSP5 (in addition to VAR2CSA DBL1-2, which was not affected by RTS,S vaccine) were also associated with protection at M3. In contrast to IgG1, stratified analysis including only RTS,S vaccinees showed that IgG3 to PE antigens LSA1 and SSP2 and MSP1 bl2, the latter two increased after RTS,S vaccination, correlated with protection. For IgG3, associations with malaria risk were only found for MSP2 FL CH150, which was not affected by RTS,S vaccination. In multi-marker analysis of M3 antibody levels, IgG1 levels to MSP1_42_ and IgG1 and IgG3 levels to MSP2 contributed significantly and positively to the components associated with malaria, whereas IgG3 levels to numerous antigens including VAR2CSA DBL1-2, MSP1 bl2 3D7, MSP5 contributed to the components in an inverse relationship, consistent with the malaria protective associations in previous results. In addition, IgG3 to pTRAMP, CelTOS, RH4.9, SSP2, and RH2 were also important variables in the components suggesting a role in malaria protection. RTS,S vaccination also decreased the levels of IgG2 to EXP1, another exposure antigen, but it increased the levels of IgG2 to VAR2CSA DBL3-4 and IgG2 and IgG4 levels to MSP5. However, these responses were not associated with protection against malaria.

The observation that a vaccine that reduces exposure to the parasite is associated with an increase in IgG1 or IgG3 levels to certain antigens, and that this increase may be associated with malaria protection, could be related to the fact that RTS,S does not result in sterile immunity, particularly during the course of primary vaccination; instead, it is a partially-effective or “leaky” PE vaccine. To explain the differences in duration of vaccine efficacy between two cohorts of diverse MTI in the Mozambican phase 2b clinical trial (57), we hypothesized that partial protection afforded by RTS,S/AS0 may stimulate protective antibodies to certain malaria target antigens, through a reduction of merozoite release from the liver, leading to attenuated BS parasitaemia (58). Partially controlled infection would result in subpatent low antigen doses that could elicit enhanced IgG production to certain antigens, which would reflect in accelerated acquisition of BS protective immunity (59). While we could not observe this effect in a past study assessing IgG responses to a different set of antigens 6 months after vaccination (59, 60), IgG1 and IgG3 data obtained here would fit with this hypothesis. Alternatively, the effect on antibody levels could be due to antigen-specific immune stimulation provided by the adjuvant. It appears unlikely that RTS,S would be inducing antibodies to CSP that cross-react with other merozoite antigens.

In addition, IgG1 to LSA1 and SSP2 and IgG4 to CelTOS and a trend for LSA1, were associated with protection in RTS,S vaccinees in multivariable analyses. Multi-marker analysis also revealed a significant contribution of IgG3 to pTRAMP, CelTOS, and SSP2 (which were increased after RTS,S vaccination) to protection. We wondered how RTS,S might benefit the response to other PE antigens and a protective effect in RTS,S vaccinees. We speculate that the anti-CSP antibodies induced by the vaccine prevent the quick invasion of the hepatocytes (61), and this could allow a longer exposure of the sporozoites to the immune system, phagocytosis and antigen presentation to T cells, facilitating the response to PE antigens, which could have a synergistic effect with anti-CSP antibodies. When stratified by age and site at M3, an interesting pattern appeared for IgG4 to these and other antigens: children with malaria in the RTS,S group had lower levels of IgG4 while children with malaria who received the comparator vaccine had higher IgG4 levels. This suggests that there might be an RTS,S effect in the association of IgG4 responses with protection.

Age and site also affected IgG4 responses, children showing higher levels than infants, and Manhiça higher levels than Kintampo. In general, levels for IgG subclasses (less evident for IgG2) increased with age and IgG4 was not an exception. In fact, a previous study showed that IgG4 levels to EBA175 increased with age (13). As for site, a lower MTI could favor IgG4 rather than high MTI because it has been shown that anti-inflammatory cytokines, such as IL-10, increase with decreasing MTI (62). IL-10 is a key cytokine inducing IgG4, further relating this subclass with anti-inflammatory, and immune regulation effects (63). Nonetheless, we acknowledge that a bias by site cannot be discarded as the design of the study had this limitation. It included mostly malaria cases in Kintampo, and mostly no malaria controls in Manhiça, most of whom were children and the few cases were mostly infants.

Some antigens showed remarkable patterns in this study. Particularly, the higher IgG_1−4_ levels to MSP5 after RTS,S immunization and their association with protection (64, 65). IgG1 to MSP1 bl2 constructs (66, 67) also increased post-vaccination and were associated with protection. Both are merozoite surface proteins and have been associated with reduced malaria, but they also have distinct features. MSP5 is a highly conserved antigen, whose function is unknown and is not essential for growth *in vitro* (68) although it has never been reported to be absent in sequenced field strains. In contrast, MSP1 bl2 is a highly polymorphic region of the N-terminal of this large protein which C-terminal end mediates the initial steps of merozoite invasion (68). MSP5 mainly induces IgG1 whereas MSP1 bl2 mainly elicits IgG3. An explanation for this is given by the conservation theory, which says that conserved antigens mainly elicit IgG1 whereas polymorphic antigens tend to elicit IgG3 (69) although this is not a consistent finding; for example, antibodies to the conserved regions of EBA175, EBA140, EBA181, are mainly IgG3 (70). Another explanation is that antibodies to structured regions or proteins are IgG1-skewed and antibodies to disordered proteins or regions are IgG3-skewed. Due to the peculiar pattern of naturally-acquired antibody responses to MSP5 and MSP1 bl2 upon RTS,S vaccination and their association with protection, their potential role in the efficacy and duration of RTS,S vaccine immunity and as adjunct or combination vaccines merit further study.

To conclude, we found that malaria protection was associated with cytophilic IgG3 levels mostly, IgG1 levels and, unexpectedly, with IgG4 responses to vaccine-unrelated antigens, specially MSP5 and MSP1 bl2, but also PE proteins, particularly in RTS,S vaccinees. These antigens are candidates to be included in multivalent and multistage second-generation vaccines that could attain higher efficacy than current products. In contrast, IgG2 responses were associated with higher risk of malaria. Our results highlight the need to assess subclass responses to malaria antigens in addition to total IgG responses since their levels and effector mechanisms are different, with subsequent impact on malaria protection. Importantly, the data of this study provides a better understanding of the interaction between NAI and RTS,S/AS01E-induced immunity. According to our results, NAI contributes to the protection afforded by RTS,S and RTS,S vaccination affects the acquisition of NAI against PE and BS antigens, increasing responses to those that are primarily associated with protection against malaria.

## Data Availability

The raw data supporting the conclusions of this manuscript will be made available by the authors, without undue reservation, to any qualified researcher. The datasets generated for this study are available on request to the corresponding author.

## Ethics Statement

This study was carried out in accordance with ICH Good Clinical Practice guidelines and the Declaration of Helsinki. Parents/guardians of infants and children gave written informed consent for participation in the study. The protocol was approved by the following ethics committees or IRBs: Kintampo Health Research Centre (KHRC) Institutional Ethics Committee (IEC), Ghana; Noguchi Memorial Institute for Medical Research IRB, Ghana; Comité Ètic d'Investigació Clínica (CEIC, Hospital Clínic, UB), Spain; Comite Nacional de Bioetica (CNBS), Mozambique; Research Ethics Committee (REC), PATH, USA.

## Author Contributions

CD, RS, and GM wrote the first draft. CD, JC, and GM conceived the study. RS, AA, IU, and CV performed database management, statistical analysis, and experimental design. AN, CJ, JC, GM, CD, DD, KA, BGy, and SO-A collected samples and data and participated in the clinical trial. MV, AJ, and IU performed the experiments. NW, ND-P, and CD coordinated the study. IU, CD, JC, GM, RA, and CV participated in the design of the analysis. DL, VC, CC, SD, DG, EA, BGa, RC, DC, JB, and LR provided antigens. JB contributed to the write up of the manuscript. All reviewed and approved the manuscript.

### Conflict of Interest Statement

The authors declare that the research was conducted in the absence of any commercial or financial relationships that could be construed as a potential conflict of interest.
