# Supplementary material for: Differential Patterns of IgG Subclass Responses to Plasmodium falciparum Antigens in Relation to Malaria Protection and RTS,S Vaccination
Source: Front Immunol. 2019 Mar 15;10:439. doi: 10.3389/fimmu.2019.00439 (PMC6428712; doi:10.3389/fimmu.2019.00439)
Supplement: Supplementary file 1 [file Data_Sheet_1.docx]

Supplementary Material

Differential patterns of IgG subclass responses to *Plasmodium falciparum* antigens in relation to malaria protection and RTS,S vaccination

Carlota Dobaño*, Rebeca Santano, Marta Vidal, Alfons Jiménez, Chenjerai Jairoce, Itziar Ubillos, David Dosoo, Ruth Aguilar, Nana Aba Williams, Nuria Díez-Padrisa, Aintzane Ayestaran, Clarissa Valim, Kwaku Poku Asante, Seth Owusu-Agyei, David Lanar, Virander Chauhan, Chetan Chitnis, Sheetij Dutta, Evelina Angov, Benoit Gamain, Ross Coppel, James G. Beeson, Linda Reiling Deepak Gaur, David Cavanagh, Ben Gyan, Augusto J. Nhabomba, Joseph J. Campo, Gemma Moncunill*

*** Correspondence:** [carlota.dobaño@isglobal.org](mailto:carlota.dobaño@isglobal.org) and [gemma.moncunill@isglobal.org](mailto:gemma.moncunill@isglobal.org)

# Supplementary Data

## Experimental Procedures

**Antibody measurements.** Antigen-coupled beads were added to a 96-well μClear® flat bottom plate (Greiner Bio-One) in multiplex (1,000 microspheres/analyte/well) resuspended in 50µL of PBS, 1% BSA, 0.05% Azide pH 7.4 (PBS-BN). Fifty µL of sample, negative or positive control were added to multiplex wells and incubated overnight at 4ºC in a shaker protected from light. Plates were washed three times with 200µL/well of wash buffer (PBS-Tween 20 0.05%) using a manual magnetic washer. Then, 100µL of biotinylated secondary antibody were added diluted in PBS-BN: anti-human IgG1 (Abcam) and anti-human IgG3 (Sigma). For IgG2 and IgG4, mouse anti-human IgG4 (Thermo) and mouse anti-human IgG2 (Thermo Fisher) were added, followed by biotinylated goat anti-mouse IgG (Sigma) in PBS-BN. All antibody incubations were performed for 60 min, at room temperature, with agitation and protected from light. Next, streptavidin-R-phycoerythrin (Sigma) in PBS-BN was added to all wells and incubated 30 min, at room temperature, with agitation and protected from light. Plates were washed as before and resuspended in 100 μL/well of PBS-BN. Plates were stored at 4°C overnight protected from light and read the next day using the Luminex xMAP® 100/200 analyser; at least 50 microspheres per analyte were acquired per sample**.**

Test samples were assayed at 4 dilutions for IgG1, IgG3 (100, 1000, 10,000 and 100,000) and 2 dilutions for IgG2 and IgG4 (50 and 500) to ensure that at least one dilution lie in the linear range of the respective standard curve, i.e. close to the highest slope between two dilution points. For IgG assays, 18 to 22 serial dilutions 1:2 of a positive control were used to perform antigen-isotype/subclass specific standard curves. The positive control consisted of a WHO Reference Reagent for anti-malaria P. falciparum human serum (NIBSC code: 10/198) at 1:50 plus a pool of plasmas from RTS,S/AS02 vaccinated children with high IgG titers against CSP at 1:100. A total of 69 different negative control samples from malaria-naïve adult donors were assayed along the study plates. Blanks were added to each plate in triplicates for quality control purposes. Sample distribution across plates was designed to ensure a balanced distribution of vaccination groups, age cohorts and time-points. Data were captured using xPonent software.

## Statistical analysis

**Data pre-processing.** The assay quality control for each antigen and plate was based on the estimation of the % coefficient of variation (CV) of the 3 blank controls and the performance of the standard curves. The standard curve for each antigen-isotype/subclass-plate was estimated using the drLumi R package flow(1), fitted in a 5-parameter logistic (5-PL) regression model, and data points logarithmically transformed. If the model did not converge, 4-PL or exponential regressions were fitted. The standard curves were visually inspected, and the percentage of plates within an analyte-isotype/subclass with CV Emax SE/Beta <15%, CV Emin SE/Beta <15%, R2 >0.95 and Model fit p value >0.05, were calculated.

To select the sample dilution in the linear part of the sigmoidal curve (antigen, isotype/subclass and plate specific), an algorithm that detects the two points with the highest slope between them was used. The slope was computed as: m = (log_10_ MFIi – log_10_ MFIi+1) / (dilution_factori – dilution_factori+1). The mean log10 MFI value of the two points was computed, and the nearest log_10_MFI of the test sample and the corresponding dilution was selected. For some antigens, IgG2 and IgG4 standard curves did not converge, as there is no appropriate positive standard control for these Ig subclasses; in these cases, we assigned the first sample dilution. The MFI measurement of the selected dilution was corrected multiplying by its corresponding dilution factor and transformed to log_10_ scale to stabilize the variance.

Blank and GST background signals were not subtracted. GST subtraction distorted and increased the variability of the data due to the lack of correlation between GST-fusion proteins and GST alone.

**Supplementary Figures and Tables**

## Supplementary Tables

| **Antigen** | **Rationale** | **Malaria life stage** | **Expression** | **Ref** |
| --- | --- | --- | --- | --- |
| SSP2 or TRAP | Exposure to sporozoite infection | Sporozoite |  | (2,3) |
| CelTOS |  |  |  | (4,5) |
| LSA1 |  | Liver |  | (6,7) |
| EXP1 | Exposure to asexual blood stage infection |  |  | (8) |
| AMA1 3D7 (FMP2.1) |  | Merozoite |  | (9,10) |
| AMA1 FVO (FMP009) |  |  |  | (9) |
| CyRPA full length |  |  |  | (11) |
| EBA140 |  |  | GST fusion | (12) |
| EBA175 region 2 PfF2 |  |  |  | (13) |
| EBA175 region 3-5 |  |  | GST fusion | (12) |
| MSP1_42_ 3D7 |  |  |  | (9,14) |
| MSP1_42_ FVO |  |  |  | (9,14) |
| MSP1 Block 2 PA17 |  |  | GST fusion | (15) |
| MSP1 Block 2 3D7 |  |  | GST fusion | (15) |
| MSP1 Block 2 MAD20 |  |  | GST fusion | (15) |
| MSP1 Block 2 RO33 |  |  | GST fusion | (15) |
| MSP1 Block 2 Wellcome |  |  | GST fusion | (15) |
| MSP1 Block 2 hybrid |  |  | GST fusion | (16) |
| MSP2 CH150 full length (5/6) Type A |  |  | GST fusion | (17) |
| MSP2 Dd2 full length (13/14) Type B |  |  | GST fusion | (17) |
| MSP3 3C |  |  |  | (17) |
| MSP3 3D7 |  |  |  | (18) |
| MSP5 |  |  |  | (19,20) |
| MSP6 |  |  | GST fusion | (21) |
| PTRAMP |  |  |  | (22) |
| P41 |  |  |  | (23) |
| RH1 |  |  |  | (24) |
| RH2 b240 |  |  |  | (25) |
| RH2 (2030) |  |  | GST fusion | (26) |
| RH4.2 |  |  | GST fusion | (27,28) |
| RH4.9 |  |  |  | (27,28) |
| RH5 |  |  |  | (11,29) |
| DBLα |  | Trophozoite |  | (30) |
| VAR2CSA DBL1-2 | Representative of maternally-transferred antibodies |  |  | (31) |
| VAR2CSA DBL3-4 |  |  |  | (32) |

**Supplementary Table 1.** Antigens and controls included in the multiplex panel.

## Supplementary Figures

**
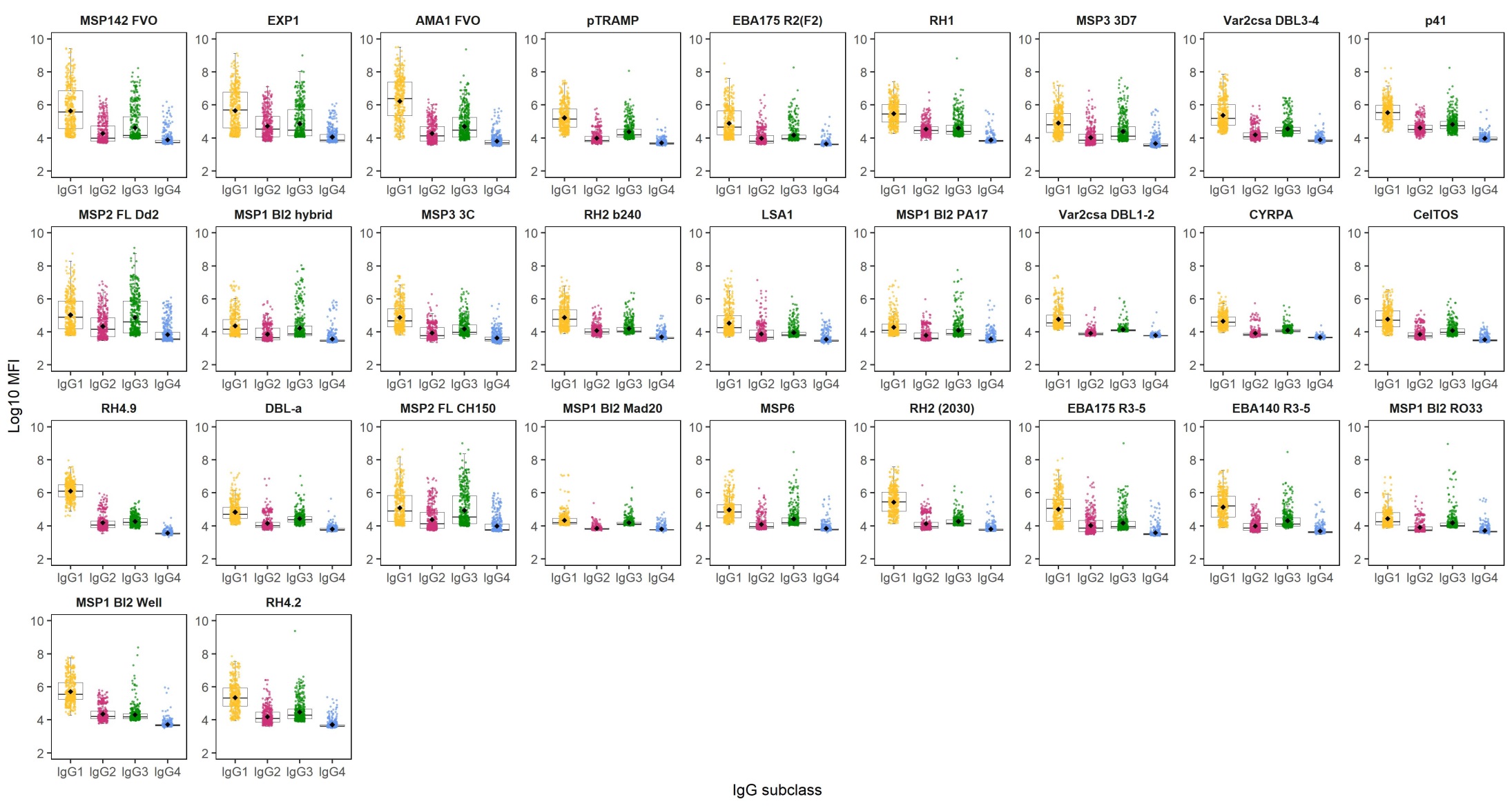
**

**Supplementary Figure 1.** Continuation from Figure 1.

**
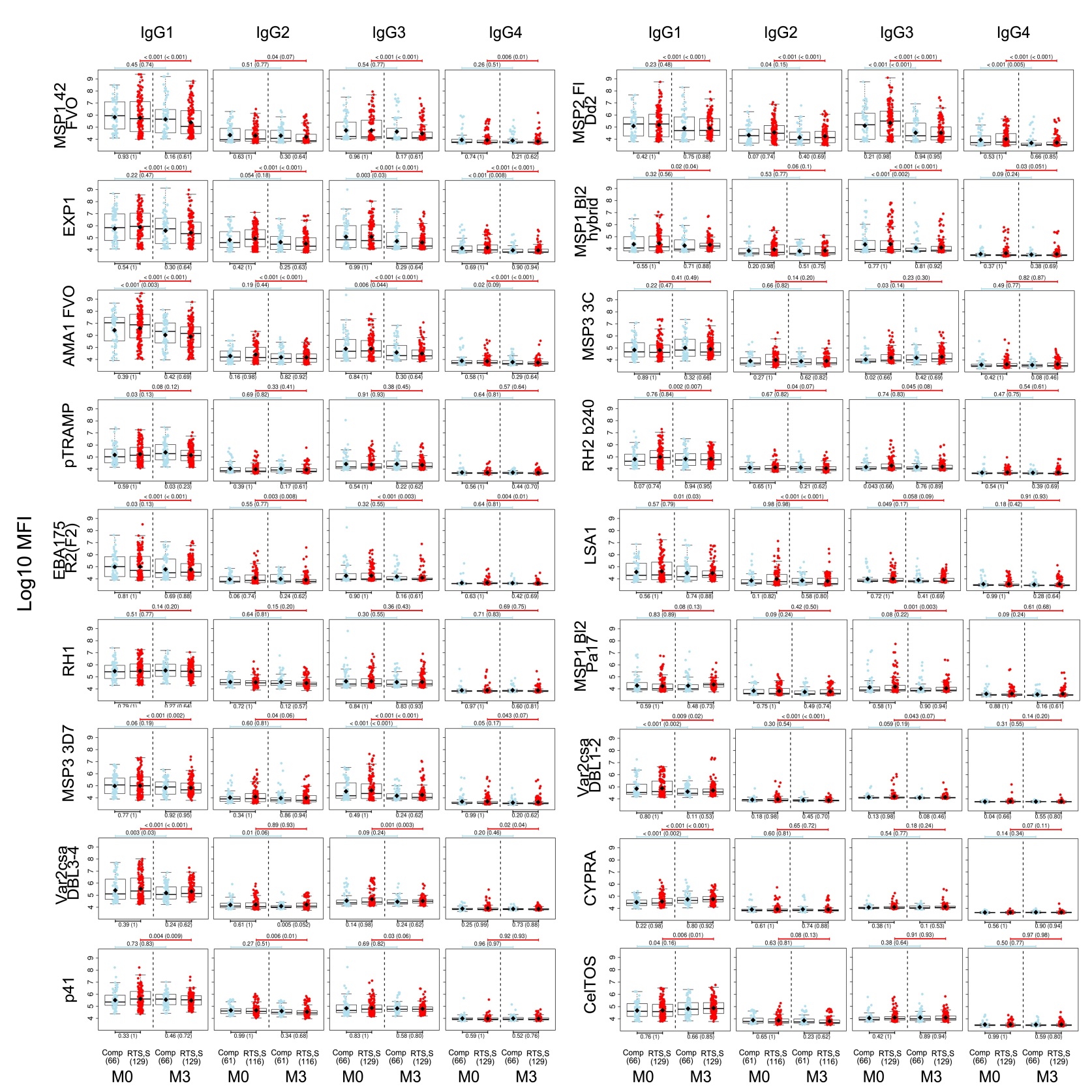
**

**Supplementary Figure 2.** Continuation from Figure 2. Continuation in next page.

**
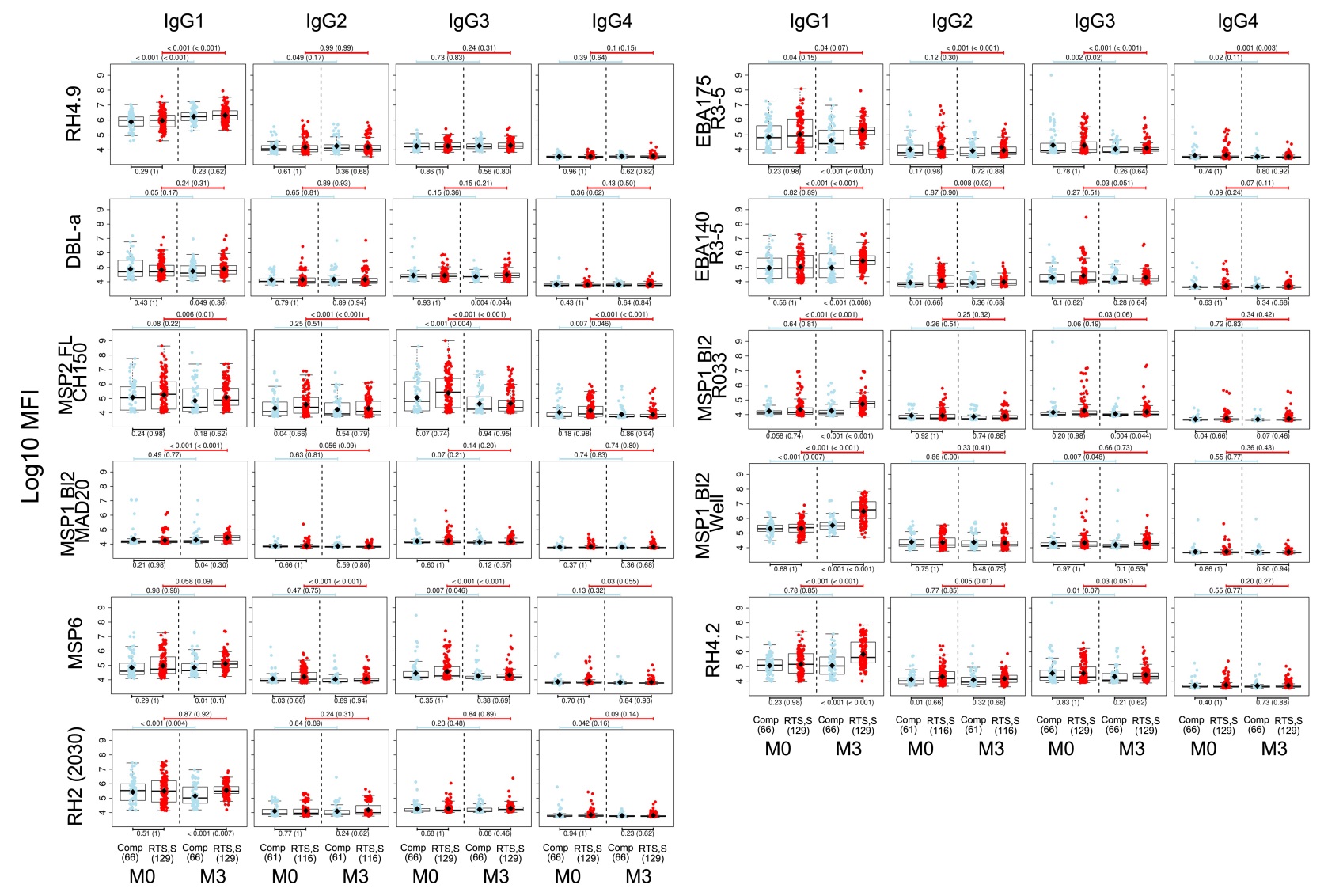
Supplementary Figure 2.** Continuation from Figure 2.

**
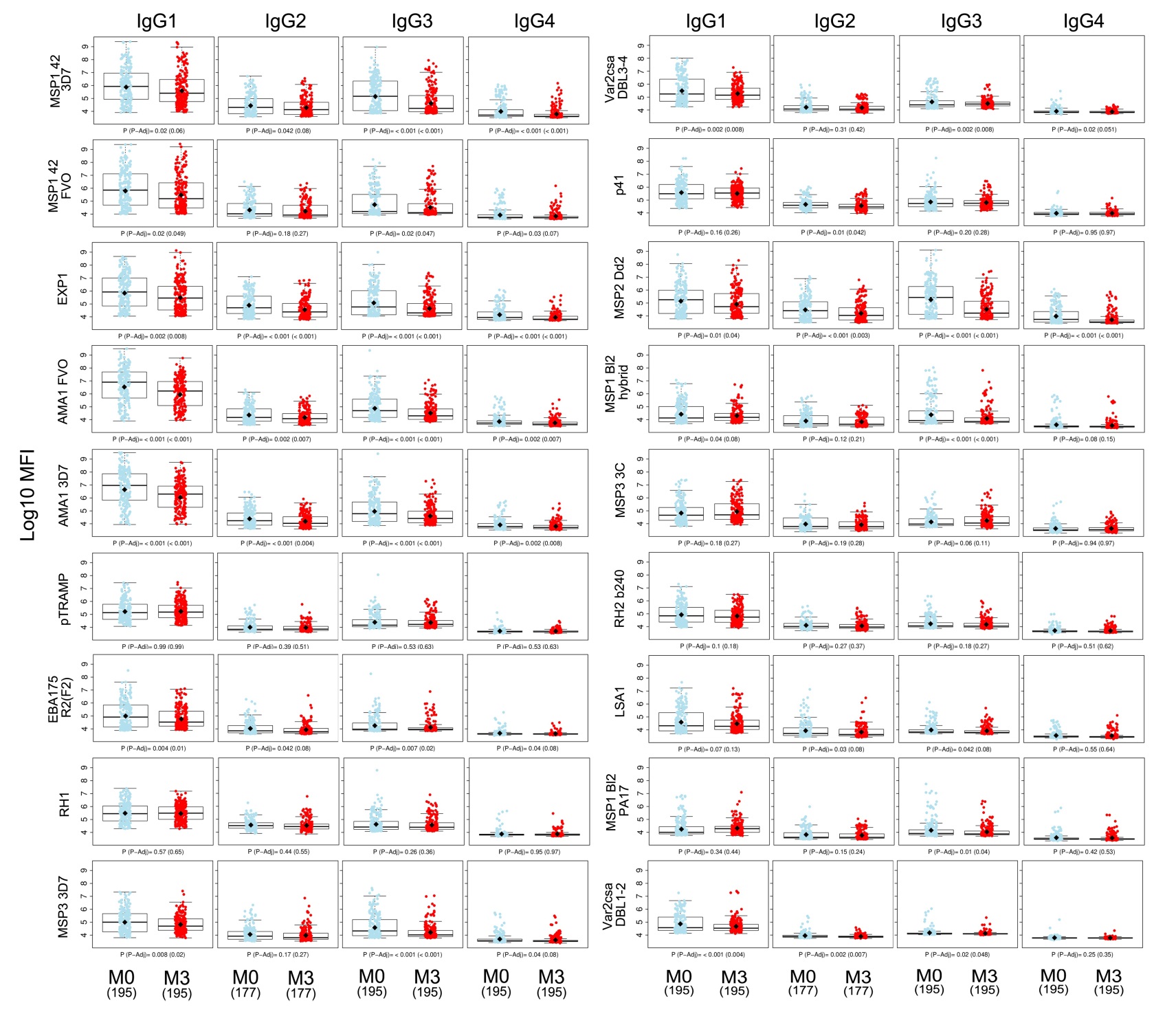
**

**Supplementary Figure 3.** IgG subclass distribution to *Plasmodium falciparum* antigens per visit group. Adjusted p-values are shown in parenthesis. Continuation in next page.

**
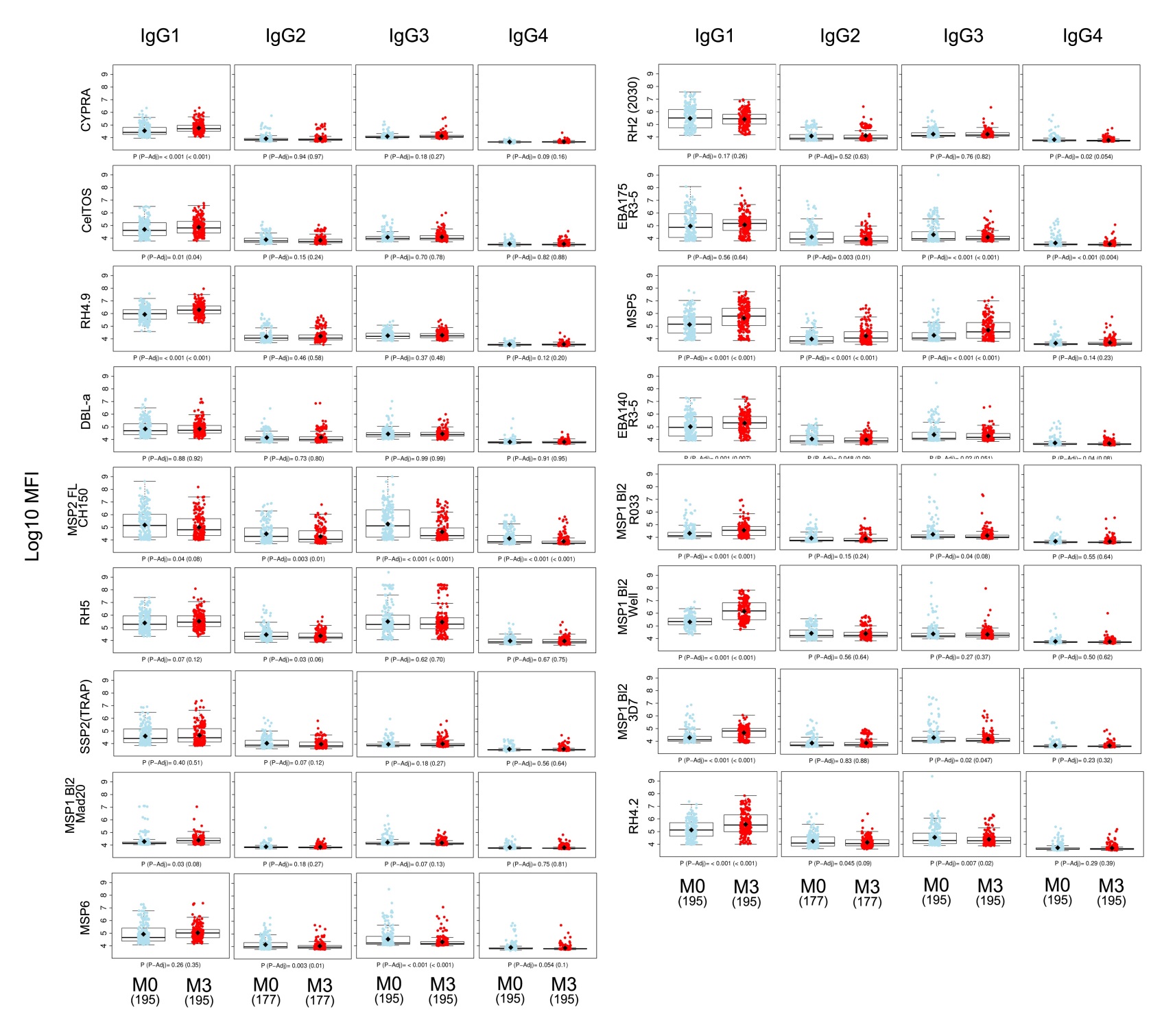
**

**Supplementary Figure 3.** IgG subclass distribution to *Plasmodium falciparum* antigens per visit group. Adjusted p-values are shown in parenthesis. Continuation from previous page.

**
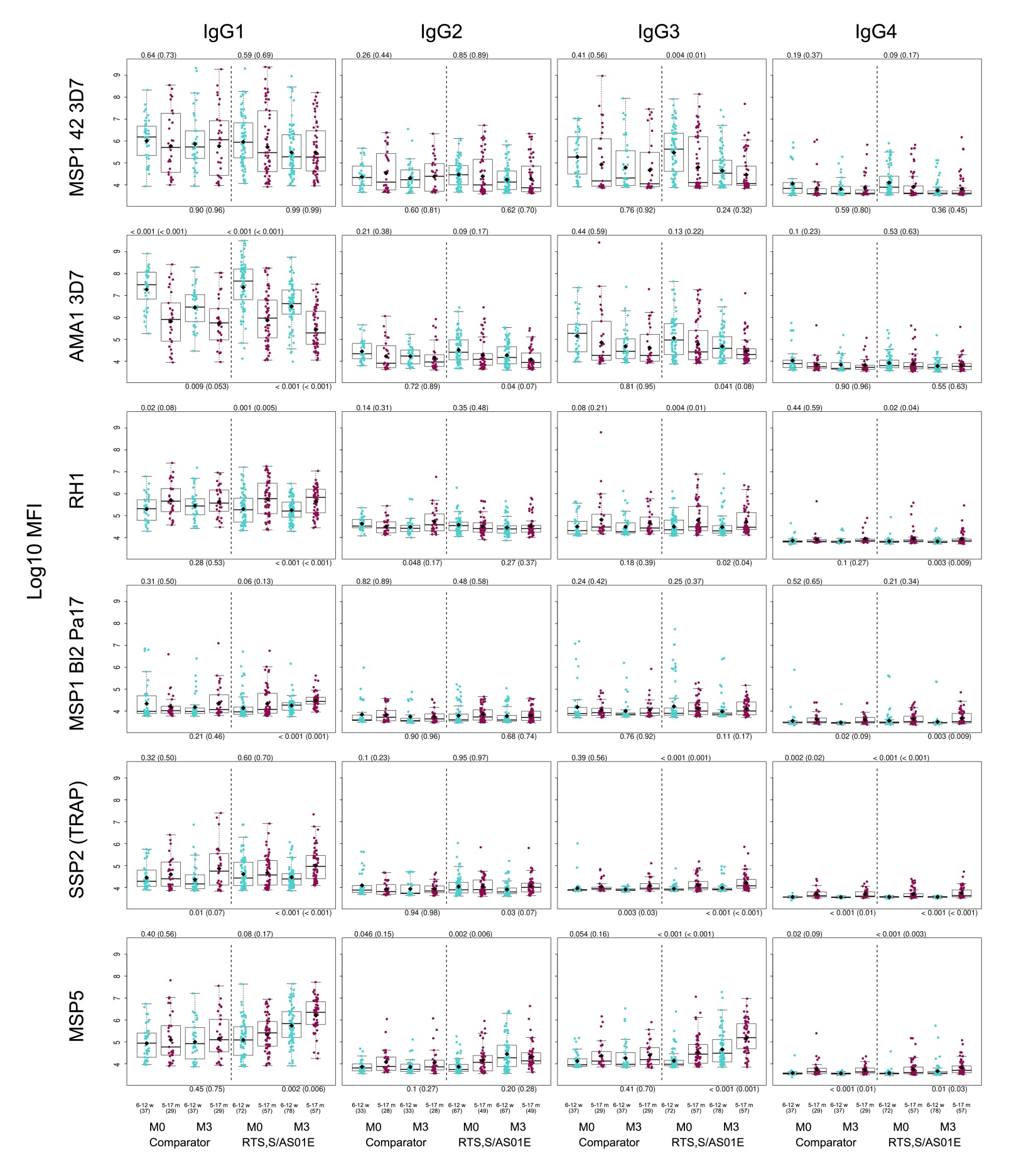
**

**Supplementary Figure 4.** IgG subclass levels to non-RTS,S *P. falciparum* antigens before and after RTS,S/AS01E vaccination stratified by age group. Adjusted p-values are shown in parenthesis.

**
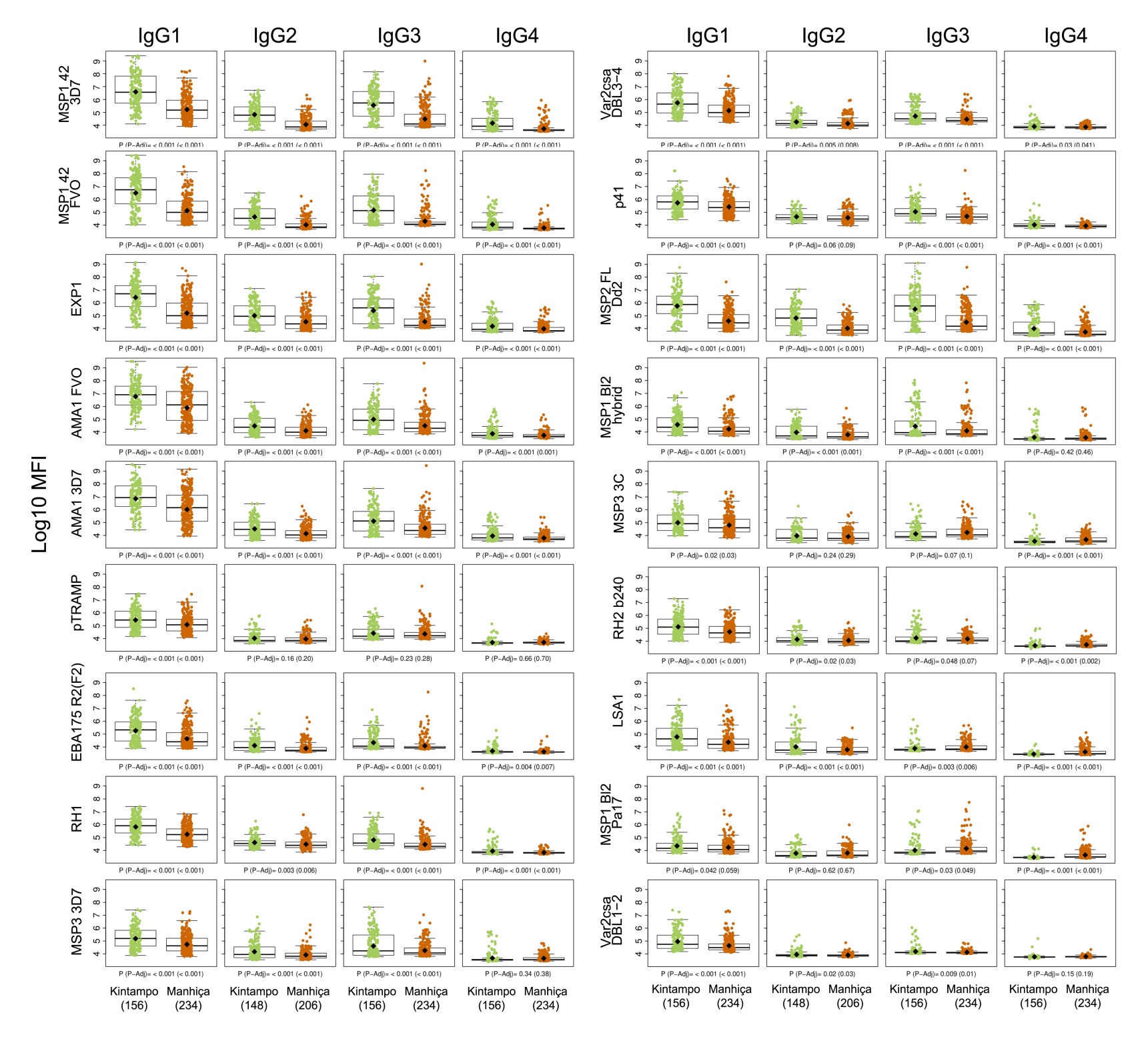
**

**Supplementary Figure 5.**  IgG subclass distribution to *Plasmodium falciparum* antigens per site. Continuation in next page.

**
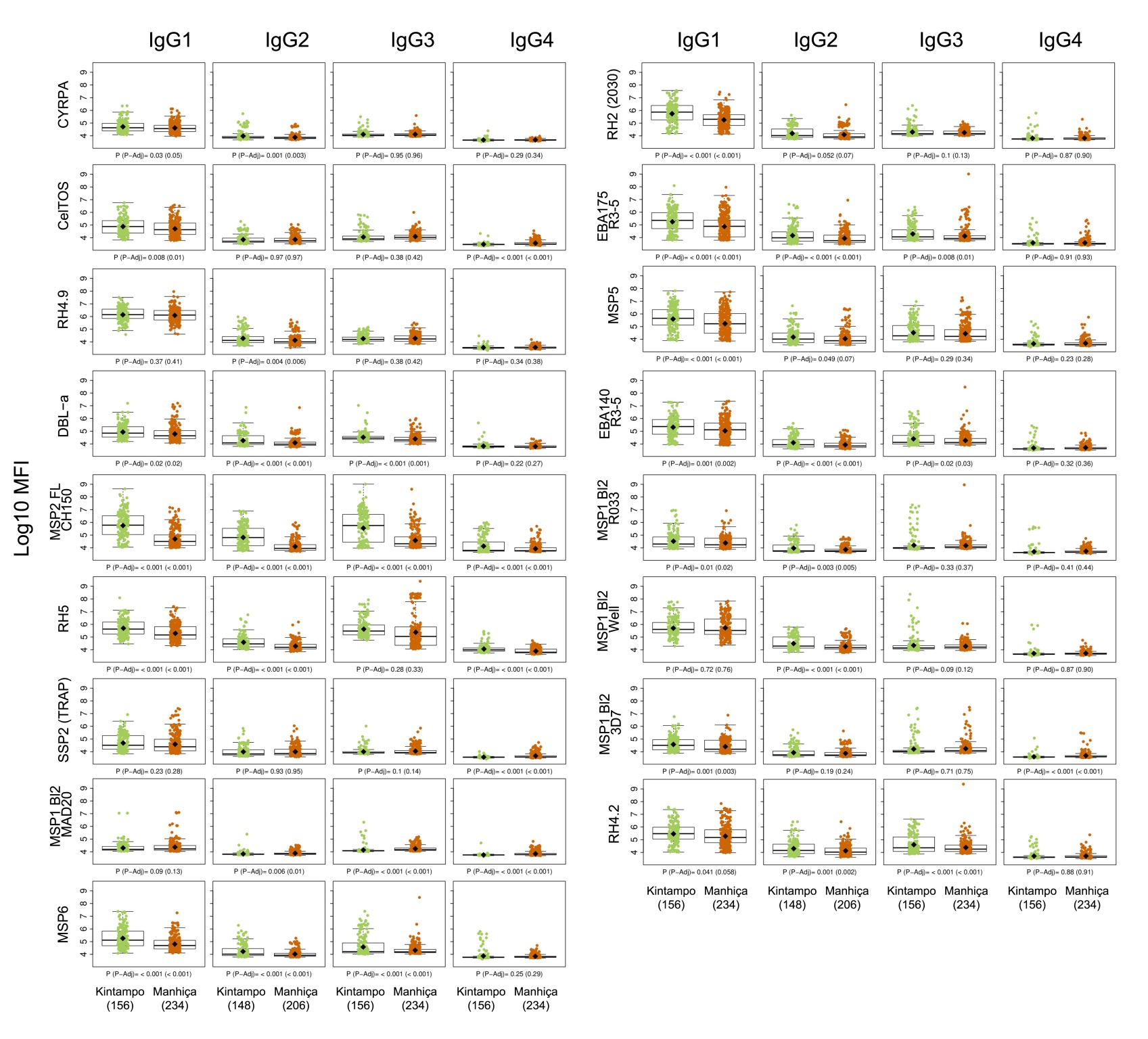
**

**Supplementary Figure 5.** IgG subclass distribution to *Plasmodium falciparum* antigens per site. Continuation from previous page.

**
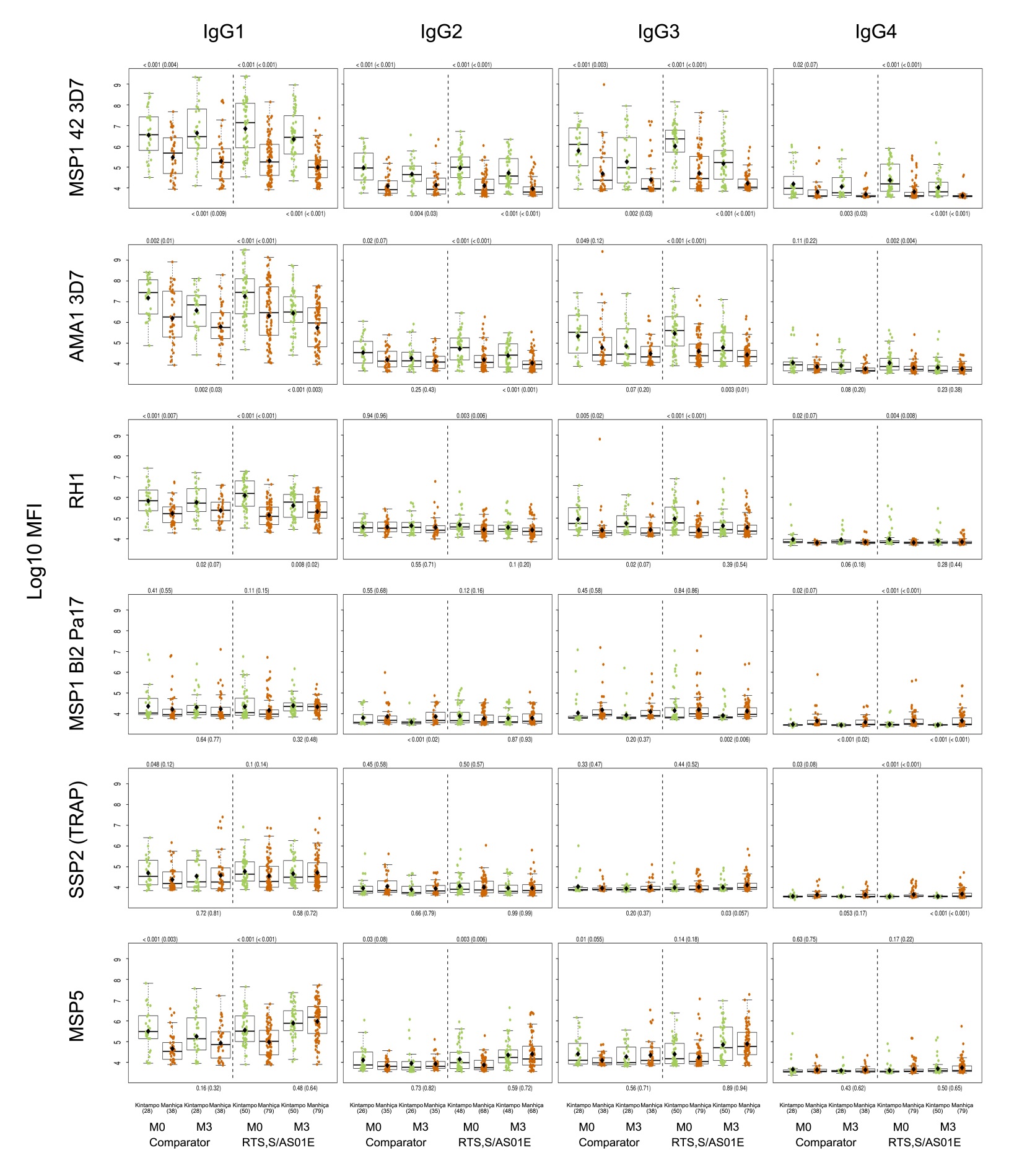
**

**Supplementary Figure 6.** IgG subclass levels to non-RTS,S *P. falciparum* antigens before and after RTS,S/AS01E vaccination stratified by site. Adjusted p-values are shown in parenthesis.

**
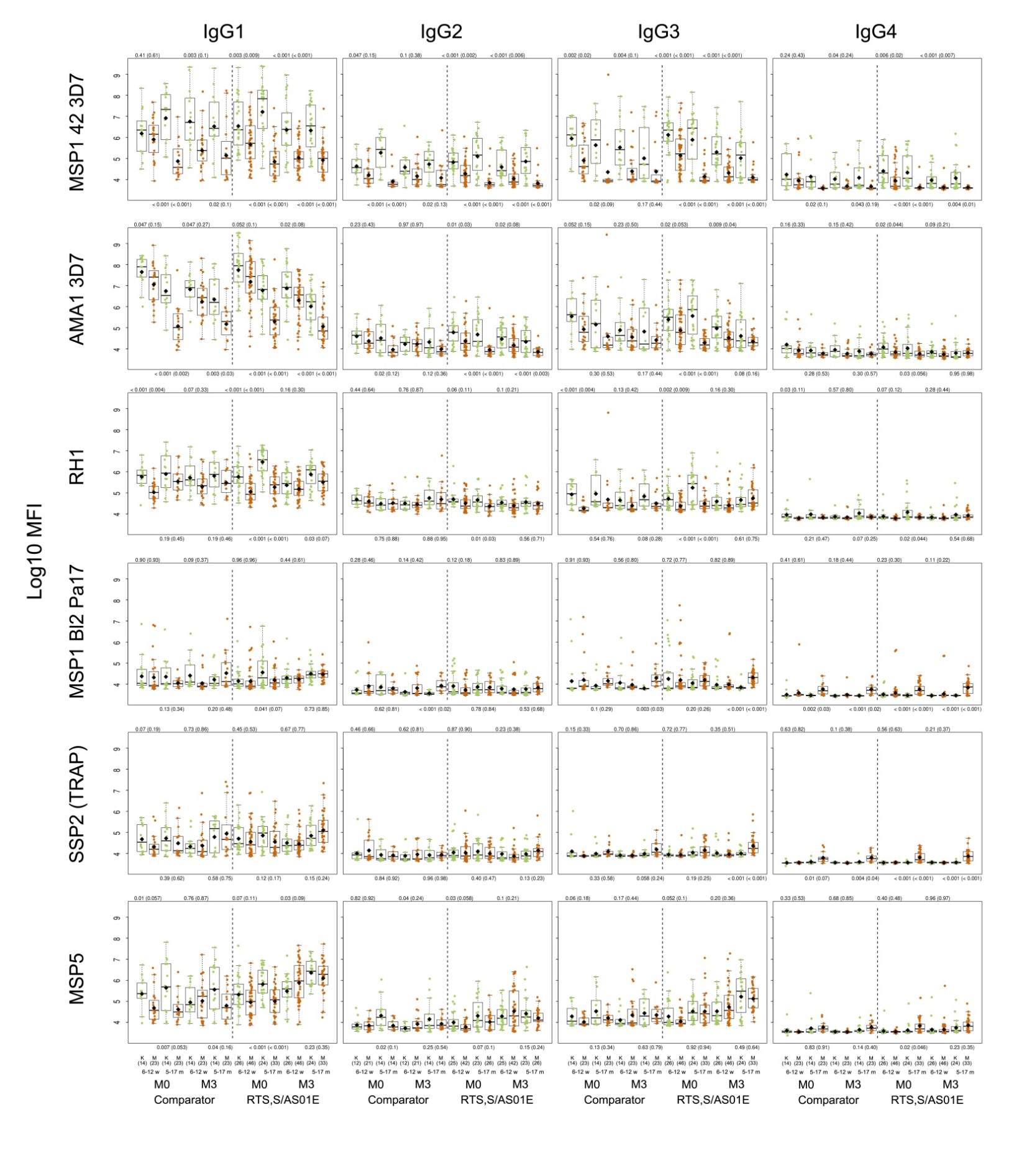
**

**Supplementary Figure 7.** IgG subclass levels to non-RTS,S *P. falciparum* antigens before and after RTS,S/AS01E vaccination stratified by age and site. K= Kintampo, M= Manhiça. Adjusted p-values are shown in parenthesis.

**
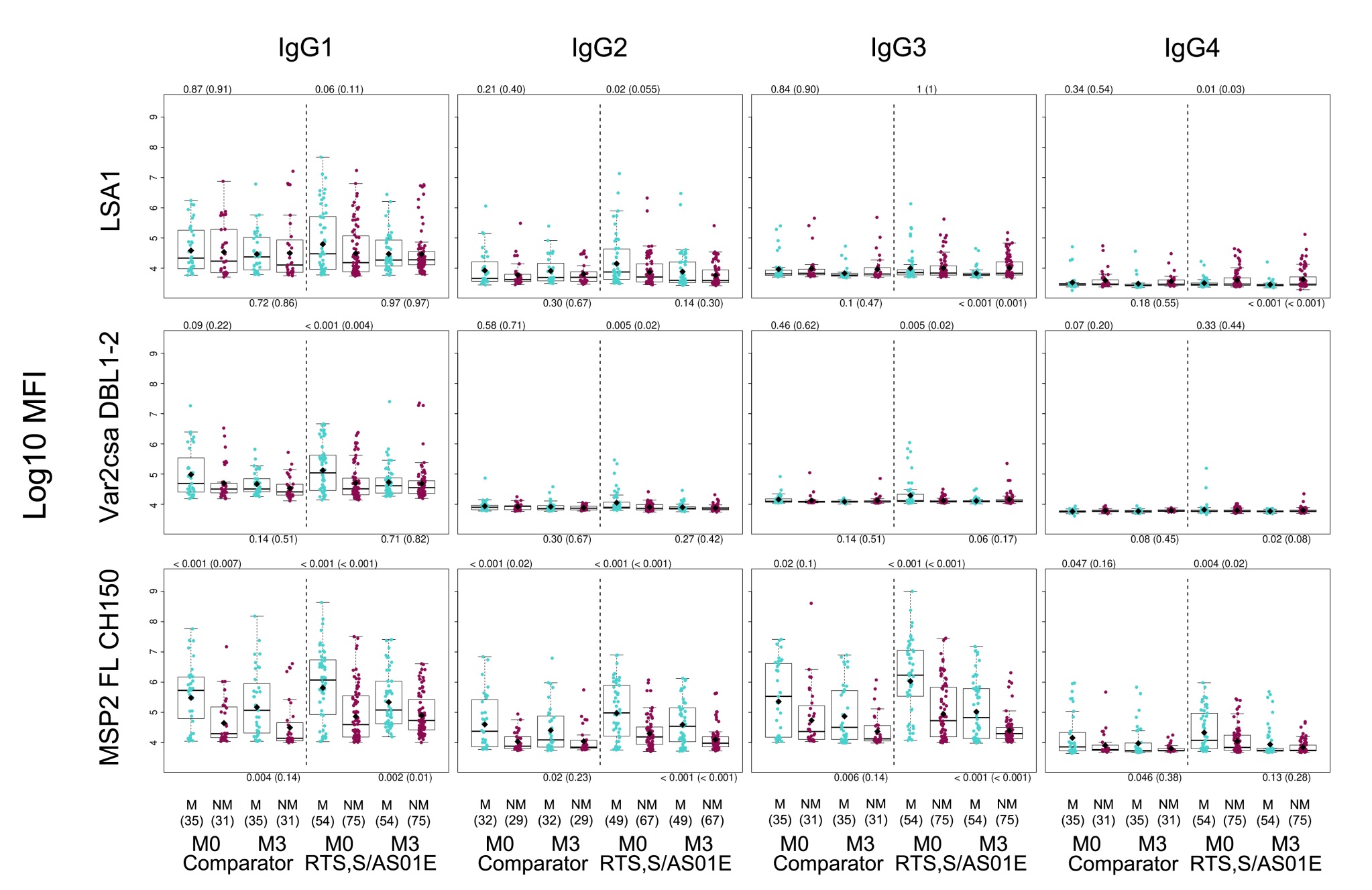
**

**Supplementary Figure 8.** Association between M3 IgG levels and malaria protection before and after RTS,S vaccination. M = Malaria, NM = No malaria. Adjusted p-values are shown in parenthesis.

**
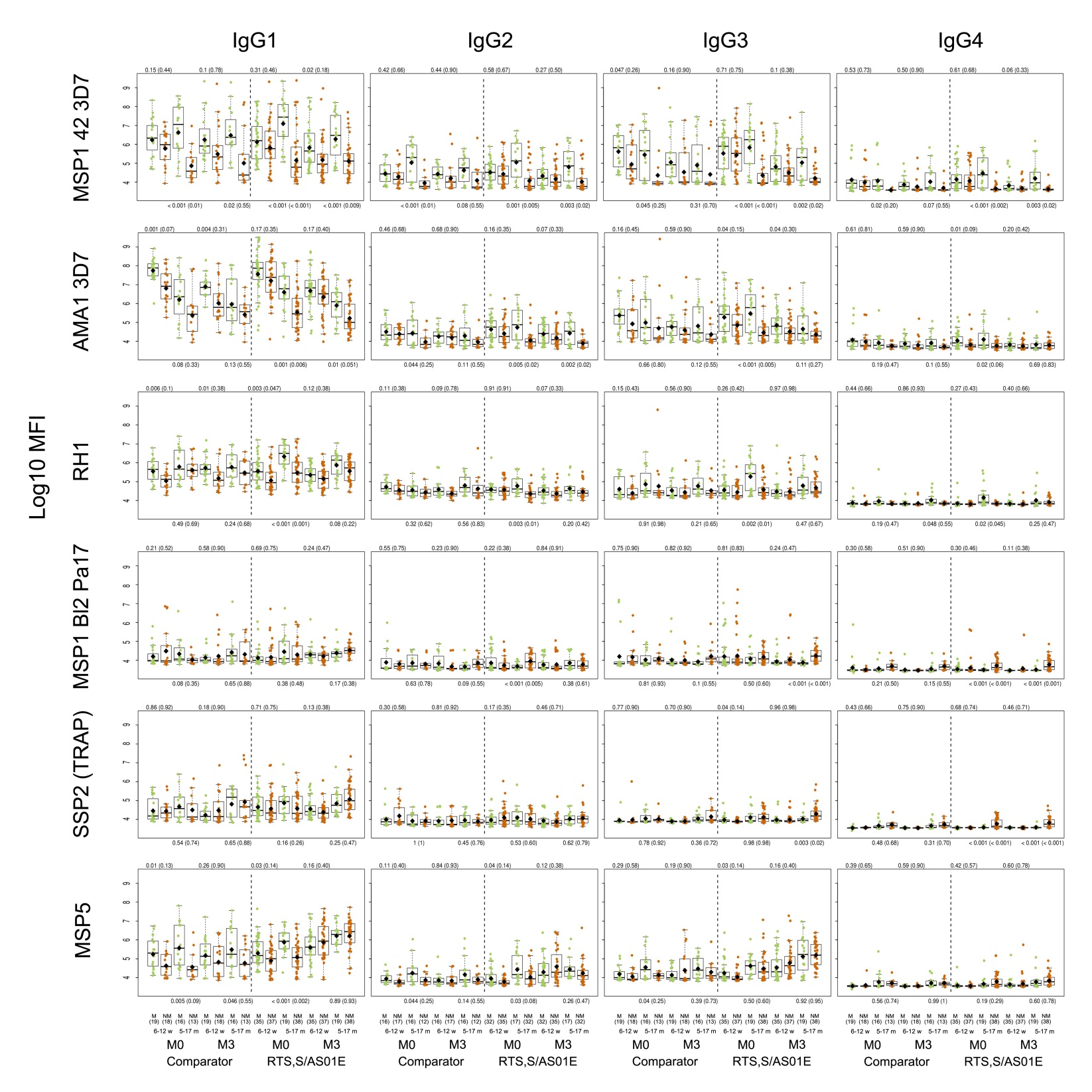
**

**Supplementary Figure 9.** IgG subclass levels to non-RTS,S *P. falciparum* antigens before and after RTS,S/AS01E vaccination stratified by malaria and age. M = malaria, NM = no malaria. Adjusted p-values are shown in parenthesis.

**
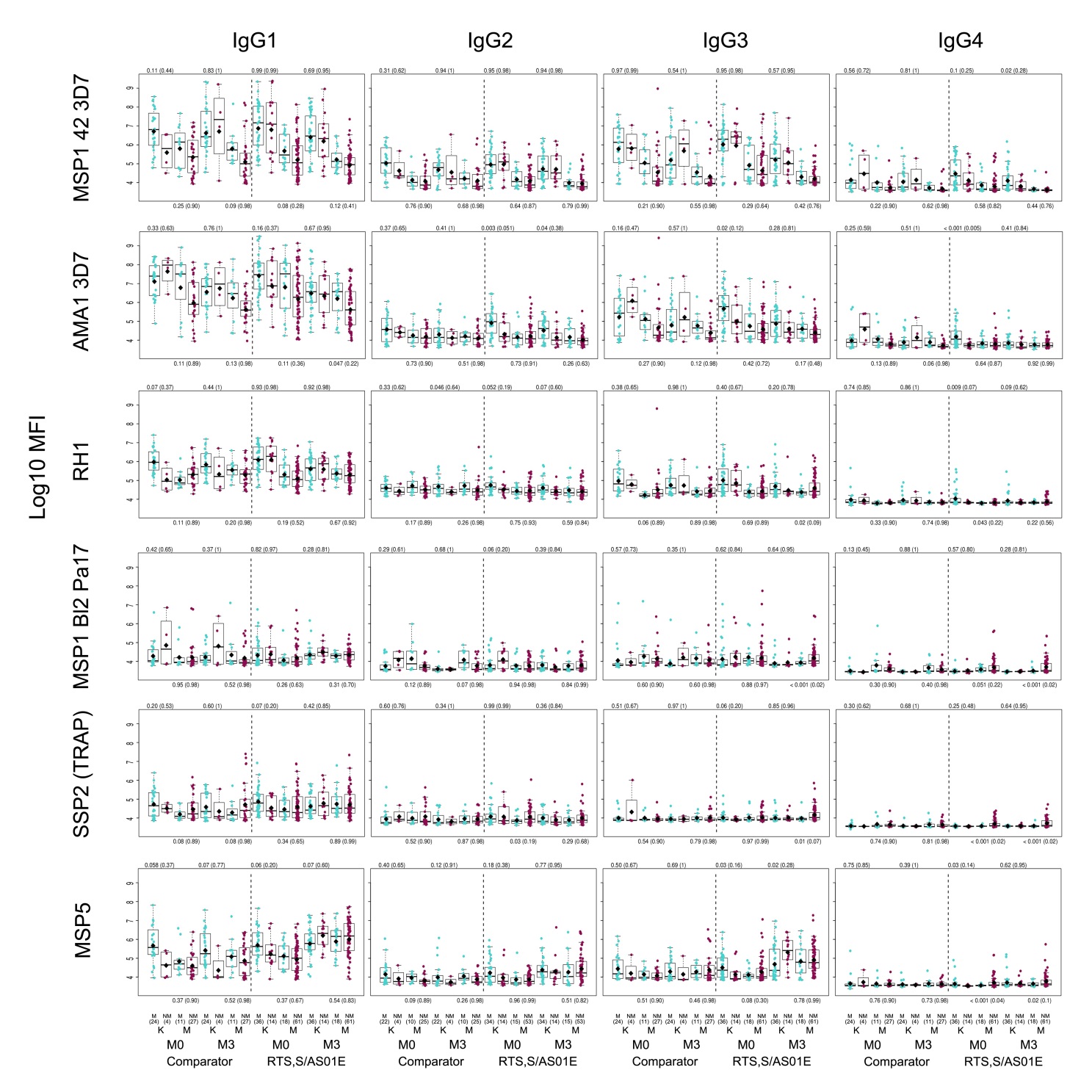
**

**Supplementary Figure 10.** IgG subclass levels to non-RTS,S *P. falciparum* antigens before and after RTS,S/AS01E vaccination stratified by malaria (M = malaria, NM = no malaria) and site (K= Kintampo, M= Manhiça). Adjusted p-values are shown in parenthesis.

**
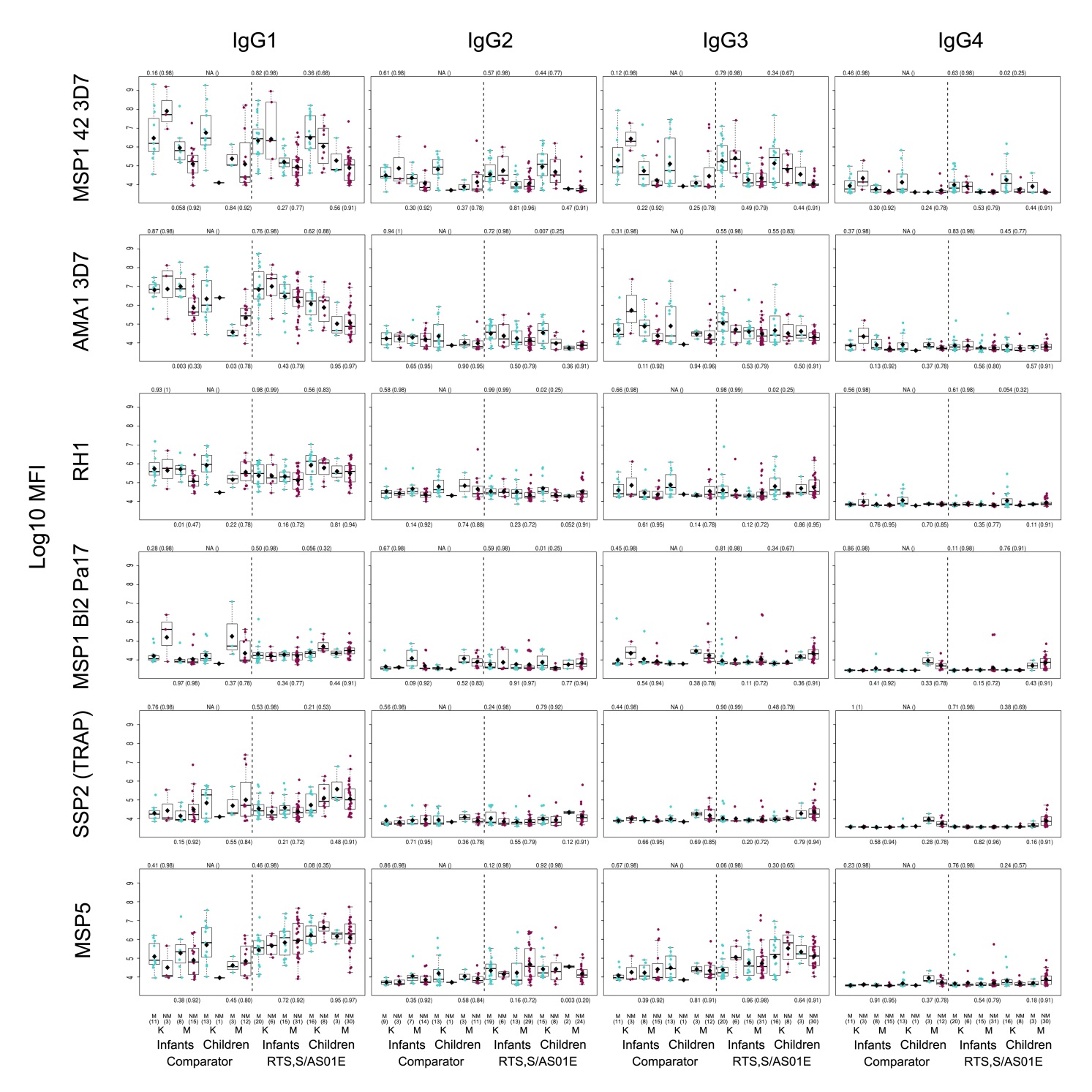
Supplementary Figure 11.** IgG subclass levels to non-RTS,S *P. falciparum* antigens after RTS,S/AS01E vaccination stratified by malaria (M = malaria, NM = no malaria), age and site (K= Kintampo, M= Manhiça) at month 3. Adjusted p-values are shown in parenthesis.


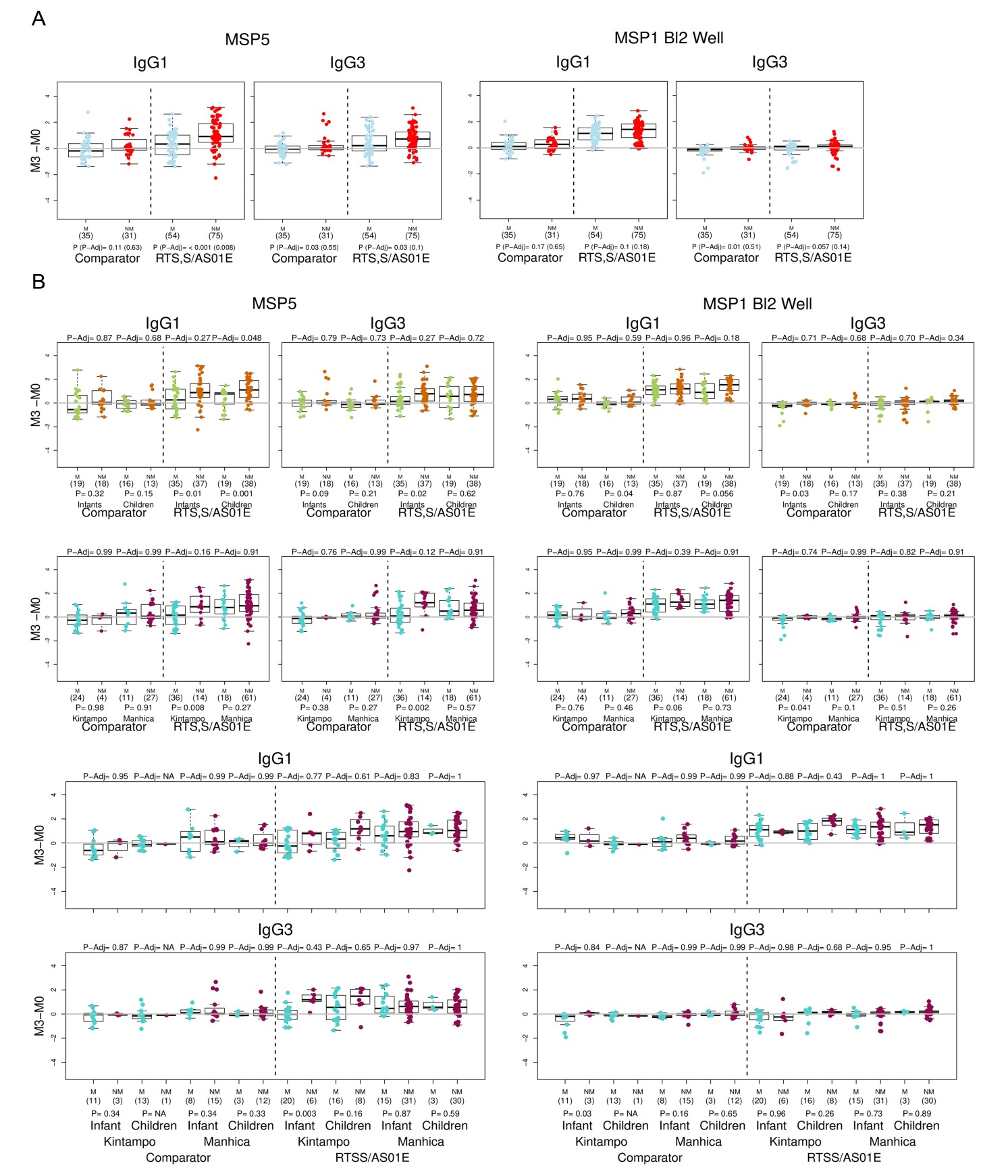


**Supplementary Figure 12**. Association between combined IgG responses and malaria protection after RTS,S vaccination. **A.** Change in levels of IgG1 and IgG3 antibodies from pre- to post-vaccination with RTS,S. **B.** Change in levels of IgG1 and IgG3 antibodies from pre- to post-vaccination with RTS,S stratified by age and site. M = Malaria, NM = No malaria. Adjusted p-values are shown in parenthesis.

## References

1. Sanz H, Aponte JJ, Harezlak J, Dong Y, Ayestaran A, Nhabomba A, Mpina M, Maurin OR, Díez-Padrisa N, Aguilar R, et al. drLumi: An open-source package to manage data, calibrate, and conduct quality control of multiplex bead-based immunoassays data analysis. *PLoS One* (2017) **12**:1–18. doi:10.1371/journal.pone.0187901

2. Khusmith S, Charoenvit Y, Kumar S, Sedegah M, Beaudoin RL, Hoffman SL. Protection against malaria by vaccination with sporozoite surface protein 2 plus CS protein. *Science* (1991) **252**:715–718.

3. Robson KJ, Hall JR, Jennings MW, Harris TJ, Marsh K, Newbold CI, Tate VE, Weatherall DJ. A highly conserved amino-acid sequence in thrombospondin, properdin and in proteins from sporozoites and blood stages of a human malaria parasite. *Nature* (1988) **335**:79–82. doi:10.1038/335079a0

4. Kusi KA, Bosomprah S, Dodoo D, Kyei-Baafour E, Dickson EK, Mensah D, Angov E, Dutta S, Sedegah M, Koram KA. Anti-sporozoite antibodies as alternative markers for malaria transmission intensity estimation. *Malar J* (2014) **13**:103. doi:10.1186/1475-2875-13-103

5. Bergmann-Leitner ES, Hosie H, Trichilo J, Deriso E, Ranallo RT, Alefantis T, Savranskaya T, Grewal P, Ockenhouse CF, Venkatesan MM, et al. Self-adjuvanting bacterial vectors expressing pre-erythrocytic antigens induce sterile protection against malaria. *Front Immunol* (2013) **4**:176. doi:10.3389/fimmu.2013.00176

6. Guerin-Marchand C, Druilhe P, Galey B, Londono A, Patarapotikul J, Beaudoin RL, Dubeaux C, Tartar A, Mercereau-Puijalon O, Langsley G. A liver-stage-specific antigen of Plasmodium falciparum characterized by gene cloning. *Nature* (1987) **329**:164–167. doi:10.1038/329164a0

7. Zhu J, Hollingdale MR. Structure of Plasmodium falciparum liver stage antigen-1. *Mol Biochem Parasitol* (1991) **48**:223–226.

8. Doolan DL, Hedstrom RC, Rogers WO, Charoenvit Y, Rogers M, de la Vega P, Hoffman SL. Identification and characterization of the protective hepatocyte erythrocyte protein 17 kDa gene of Plasmodium yoelii, homolog of Plasmodium falciparum exported protein 1. *J Biol Chem* (1996) **271**:17861–17868.

9. Angov E, Hillier CJ, Kincaid RL, Lyon JA. Heterologous protein expression is enhanced by harmonizing the codon usage frequencies of the target gene with those of the expression host. *PLoS One* (2008) **3**:e2189. doi:10.1371/journal.pone.0002189

10. Kocken CHM, Withers-Martinez C, Dubbeld MA, van der Wel A, Hackett F, Valderrama A, Blackman MJ, Thomas AW. High-level expression of the malaria blood-stage vaccine candidate Plasmodium falciparum apical membrane antigen 1 and induction of antibodies that inhibit erythrocyte invasion. *Infect Immun* (2002) **70**:4471–4476.

11. Reddy KS, Amlabu E, Pandey AK, Mitra P, Chauhan VS, Gaur D. Multiprotein complex between the GPI-anchored CyRPA with PfRH5 and PfRipr is crucial for Plasmodium falciparum erythrocyte invasion. *Proc Natl Acad Sci U S A* (2015) **112**:1179–1184. doi:10.1073/pnas.1415466112

12. Persson KEM, Fowkes FJI, McCallum FJ, Gicheru N, Reiling L, Richards JS, Wilson DW, Lopaticki S, Cowman AF, Marsh K, et al. Erythrocyte-binding antigens of Plasmodium falciparum are targets of human inhibitory antibodies and function to evade naturally acquired immunity. *J Immunol* (2013) **191**:785–794. doi:10.4049/jimmunol.1300444

13. Pandey KC, Singh S, Pattnaik P, Pillai CR, Pillai U, Lynn A, Jain SK, Chitnis CE. Bacterially expressed and refolded receptor binding domain of Plasmodium falciparum EBA-175 elicits invasion inhibitory antibodies. *Mol Biochem Parasitol* (2002) **123**:23–33.

14. Angov E, Aufiero BM, Turgeon AM, Van Handenhove M, Ockenhouse CF, Kester KE, Walsh DS, McBride JS, Dubois M-C, Cohen J, et al. Development and pre-clinical analysis of a Plasmodium falciparum Merozoite Surface Protein-1(42) malaria vaccine. *Mol Biochem Parasitol* (2003) **128**:195–204.

15. Cavanagh DR, McBride JS. Antigenicity of recombinant proteins derived from Plasmodium falciparum merozoite surface protein 1. *Mol Biochem Parasitol* (1997) **85**:197–211.

16. Cowan GJM, Creasey AM, Dhanasarnsombut K, Thomas AW, Remarque EJ, Cavanagh DR. A malaria vaccine based on the polymorphic block 2 region of MSP-1 that elicits a broad serotype-spanning immune response. *PLoS One* (2011) **6**:e26616. doi:10.1371/journal.pone.0026616

17. Metzger WG, Okenu DMN, Cavanagh DR, Robinson J V, Bojang KA, Weiss HA, McBride JS, Greenwood BM, Conway DJ. Serum IgG3 to the Plasmodium falciparum merozoite surface protein 2 is strongly associated with a reduced prospective risk of malaria. *Parasite Immunol* (2003) **25**:307–312.

18. Imam M, Singh S, Kaushik NK, Chauhan VS. Plasmodium falciparum merozoite surface protein 3: oligomerization, self-assembly, and heme complex formation. *J Biol Chem* (2014) **289**:3856–3868. doi:10.1074/jbc.M113.520239

19. Black CG, Wang L, Hibbs AR, Werner E, Coppel RL. Identification of the Plasmodium chabaudi homologue of merozoite surface proteins 4 and 5 of Plasmodium falciparum. *Infect Immun* (1999) **67**:2075–2081. Available at: https://www.ncbi.nlm.nih.gov/pubmed/10225857

20. Black CG, Barnwell JW, Huber CS, Galinski MR, Coppel RL. The Plasmodium vivax homologues of merozoite surface proteins 4 and 5 from Plasmodium falciparum are expressed at different locations in the merozoite. *Mol Biochem Parasitol* (2002) **120**:215–224. doi:https://doi.org/10.1016/S0166-6851(01)00458-3

21. Hill DL, Wilson DW, Sampaio NG, Eriksson EM, Ryg-Cornejo V, Harrison GLA, Uboldi AD, Robinson LJ, Beeson JG, Siba P, et al. Merozoite Antigens of Plasmodium falciparum Elicit Strain-Transcending Opsonizing Immunity. *Infect Immun* (2016) **84**:2175–2184. doi:10.1128/IAI.00145-16

22. Siddiqui FA, Dhawan S, Singh S, Singh B, Gupta P, Pandey A, Mohmmed A, Gaur D, Chitnis CE. A thrombospondin structural repeat containing rhoptry protein from Plasmodium falciparum mediates erythrocyte invasion. *Cell Microbiol* (2013) **15**:1341–1356. doi:10.1111/cmi.12118

23. Taechalertpaisarn T, Crosnier C, Bartholdson SJ, Hodder AN, Thompson J, Bustamante LY, Wilson DW, Sanders PR, Wright GJ, Rayner JC, et al. Biochemical and Functional Analysis of Two Plasmodium falciparum Blood-Stage 6-Cys Proteins: P12 and P41. *PLoS One* (2012) **7**:e41937. Available at: https://doi.org/10.1371/journal.pone.0041937

24. Gaur D, Mayer DCG, Miller LH. Parasite ligand-host receptor interactions during invasion of erythrocytes by Plasmodium merozoites. *Int J Parasitol* (2004) **34**:1413–1429. doi:10.1016/j.ijpara.2004.10.010

25. Sahar T, Reddy KS, Bharadwaj M, Pandey AK, Singh S, Chitnis CE, Gaur D. Plasmodium falciparum reticulocyte binding-like homologue protein 2 (PfRH2) is a key adhesive molecule involved in erythrocyte invasion. *PLoS One* (2011) **6**:e17102. doi:10.1371/journal.pone.0017102

26. Reiling L, Richards JS, Fowkes FJI, Barry AE, Triglia T, Chokejindachai W, Michon P, Tavul L, Siba PM, Cowman AF, et al. Evidence that the erythrocyte invasion ligand PfRh2 is a target of protective immunity against Plasmodium falciparum malaria. *J Immunol* (2010) **185**:6157–6167. doi:10.4049/jimmunol.1001555

27. Tham W-H, Wilson DW, Reiling L, Chen L, Beeson JG, Cowman AF. Antibodies to reticulocyte binding protein-like homologue 4 inhibit invasion of Plasmodium falciparum into human erythrocytes. *Infect Immun* (2009) **77**:2427–2435. doi:10.1128/IAI.00048-09

28. Reiling L, Richards JS, Fowkes FJI, Wilson DW, Chokejindachai W, Barry AE, Tham W-H, Stubbs J, Langer C, Donelson J, et al. The Plasmodium falciparum Erythrocyte Invasion Ligand Pfrh4 as a Target of Functional and Protective Human Antibodies against Malaria. *PLoS One* (2012) **7**:e45253. Available at: https://doi.org/10.1371/journal.pone.0045253

29. Reddy KS, Pandey AK, Singh H, Sahar T, Emmanuel A, Chitnis CE, Chauhan VS, Gaur D. Bacterially expressed full-length recombinant Plasmodium falciparum RH5 protein binds erythrocytes and elicits potent strain-transcending parasite-neutralizing antibodies. *Infect Immun* (2014) **82**:152–164. doi:10.1128/IAI.00970-13

30. Mayor A, Rovira-Vallbona E, Srivastava A, Sharma SK, Pati SS, Puyol L, Quinto L, Bassat Q, Machevo S, Mandomando I, et al. Functional and immunological characterization of a Duffy binding-like alpha domain from Plasmodium falciparum erythrocyte membrane protein 1 that mediates rosetting. *Infect Immun* (2009) **77**:3857–3863. doi:10.1128/IAI.00049-09

31. Chene A, Gangnard S, Dechavanne C, Dechavanne S, Srivastava A, Tetard M, Hundt S, Leroy O, Havelange N, Viebig NK, et al. Down-selection of the VAR2CSA DBL1-2 expressed in E. coli as a lead antigen for placental malaria vaccine development. *NPJ vaccines* (2018) **3**:28. doi:10.1038/s41541-018-0064-6

32. Gangnard S, Lewit-Bentley A, Dechavanne S, Srivastava A, Amirat F, Bentley GA, Gamain B. Structure of the DBL3X-DBL4epsilon region of the VAR2CSA placental malaria vaccine candidate: insight into DBL domain interactions. *Sci Rep* (2015) **5**:14868. doi:10.1038/srep14868
